# Resveratrol Reverses Functional Chagas Heart Disease in Mice

**DOI:** 10.1371/journal.ppat.1005947

**Published:** 2016-10-27

**Authors:** Glaucia Vilar-Pereira, Vitor C. Carneiro, Hilton Mata-Santos, Amanda R. R. Vicentino, Isalira P. Ramos, Naira L. L. Giarola, Daniel F. Feijó, José R. Meyer-Fernandes, Heitor A. Paula-Neto, Emiliano Medei, Marcelo T. Bozza, Joseli Lannes-Vieira, Claudia N. Paiva

**Affiliations:** 1 Fundação Oswaldo Cruz, Instituto Oswaldo Cruz, Laboratório de Biologia das Interações, Rio de Janeiro, RJ, Brazil; 2 Universidade Federal do Rio de Janeiro (UFRJ), Instituto de Bioquímica Médica Leopoldo de Meis, Programa de Biologia Molecular e Tecnologia, Rio de Janeiro, RJ, Brazil; 3 UFRJ, Instituto de Microbiologia Paulo de Góes, Departamento de Imunologial, Rio de Janeiro, RJ, Brazil; 4 UFRJ, Faculdade de Farmácia, Departamento de Análises Clínicas e Toxicológicas, Rio de Janeiro, RJ, Brazil; 5 UFRJ, Hospital Universitário Clementino Fraga Filho, Departamento de Radiologia, Rio de Janeiro, RJ, Brazil; 6 UFRJ, Instituto de Biofísica Carlos Chagas Filho, Rio de Janeiro, RJ, Brazil; 7 UFRJ, Centro Nacional de Biologia Estrutural e Bioimagem, Rio de Janeiro, RJ, Brazil; 8 UFRJ, Instituto de Bioquímica Médica Leopoldo de Meis, Rio de Janeiro, RJ, Brazil; 9 Instituto Nacional de Ciência e Tecnologia da Biologia Estrutural e Bioimagem, Rio de Janeiro, RJ, Brazil; 10 UFRJ, Faculdade de Farmácia, Departamento de Fármacos, Rio de Janeiro, RJ, Brazil; Universidade Federal de Minas Gerais, BRAZIL

## Abstract

Chronic chagasic cardiomyopathy (CCC) develops years after acute infection by *Trypanosoma cruzi* and does not improve after trypanocidal therapy, despite reduction of parasite burden. During disease, the heart undergoes oxidative stress, a potential causative factor for arrhythmias and contractile dysfunction. Here we tested whether antioxidants/ cardioprotective drugs could improve cardiac function in established Chagas heart disease. We chose a model that resembles B1-B2 stage of human CCC, treated mice with resveratrol and performed electrocardiography and echocardiography studies. Resveratrol reduced the prolonged PR and QTc intervals, increased heart rates and reversed sinus arrhythmia, atrial and atrioventricular conduction disorders; restored a normal left ventricular ejection fraction, improved stroke volume and cardiac output. Resveratrol activated the AMPK-pathway and reduced both ROS production and heart parasite burden, without interfering with vascularization or myocarditis intensity. Resveratrol was even capable of improving heart function of infected mice when treatment was started late after infection, while trypanocidal drug benznidazole failed. We attempted to mimic resveratrol’s actions using metformin (AMPK-activator) or tempol (SOD-mimetic). Metformin and tempol mimicked the beneficial effects of resveratrol on heart function and decreased lipid peroxidation, but did not alter parasite burden. These results indicate that AMPK activation and ROS neutralization are key strategies to induce tolerance to Chagas heart disease. Despite all tissue damage observed in established Chagas heart disease, we found that a physiological dysfunction can still be reversed by treatment with resveratrol, metformin and tempol, resulting in improved heart function and representing a starting point to develop innovative therapies in CCC.

## Author Summary

Protection against functional damage, i.e. disease tolerance, is a successful strategy against pathogens the immune system fails to eliminate. In Chagas disease, *Trypanosoma cruzi* infects the heart and many years after parasite burden is reduced to a minimum by the immune response, a cardiomyopathy ensues, often accompanied by electrical abnormalities and occasionally progressing to heart failure. Most attempted therapies targeted parasite elimination, failing to protect against progression of heart disease. Here we attempted to treat Chagas heart disease in mice with cardioprotectors/ antioxidants, since the parasite is known to promote tissue damage by oxidative stress. Our results show this strategy can partially reverse functional Chagas heart disease even when started late after disease onset, opening a new perspective for Chagas disease therapy.

## Introduction

Protection against functional damage (i.e., disease tolerance) represents a strategy that allows hosts to survive infection at minimum cost [1]. This strategy is successful against diseases caused by pathogens the immune system cannot eliminate and explains why individuals with similar pathogen burdens can present varying disease gravities [2]. The mechanisms underlying disease tolerance include activation of molecular pathways that are involved in tissue repair, fuel-sensing/energy production, and antioxidant defenses [1].

Activation of SIRT1 and AMPK fuel-sensing pathways contributes to cardioprotection [3]. Drugs that activate these pathways, such as resveratrol and metformin, can extend the healthspan of most organisms. Resveratrol, a polyphenol present in grapes, decreases ischemia-reperfusion injury of the heart, prevents atherosclerosis and interferes with Ca^2+^ handling by cardiomyocytes [4, 5], restoring heart contractility in cardiomyopathies and acting as an anti-arrhythmic drug [6–9]. Some of its cardioprotective effects have been attributed to its antioxidant properties, exerted by activation of the antioxidant-defense gene Nrf2, increased expression of mitochondrial SOD2, inhibition of NOX2 and NOX4 expression, and direct ROS scavenging [4, 10].

The protozoan *Trypanosoma cruzi* infects many tissues, including the heart, and causes Chagas disease in humans. Shortly after infection, the parasite proliferates, causing acute systemic inflammation. After this initial stage, the host’s adaptive immune system controls parasite burden and patients progress to a non-symptomatic stage, but the parasite is never eliminated. Most patients remain non-symptomatic, but 30% develop heart dysfunctional disease, which affect millions of people worldwide [11, 12].

Much as observed in humans, the hearts of rats and mice undergo severe oxidative stress in the course of experimental Chagas disease. Both NOX2 [13] and mitochondrial derived-ROS [14] contribute to produce oxidative damage in Chagas heart disease. Neutralization of oxidative stress since day 0 of infection prevents chronic contractile dysfunction [15], but once established, no treatment has been shown to reverse heart dysfunction in Chagas disease. Several attempts to reverse the loss of heart contractility and arrhythmias in CCC targeted either parasite burden or immune response, obtaining little success [15–20]. Recently, the BENEFIT clinical trial on the effects of the trypanocidal drug benznidazole on CCC failed to prevent cardiac deterioration, despite greatly reducing parasite load [21]. Treatment to CCC remains restricted to those transposed from other cardiomyopathies, doing little to decrease morbidity. The current paradigm dictates that structural damage to heart at the chronic stage impairs its function [22] and most attempts to reverse disease aim at re-building the heart with stem cells [23].

We have previously shown that oxidative stress fuels *T*. *cruzi* acute infection [24, 25]. Although a number of cardiomyopathies are known to be dependent on oxidative stress [26], the case for Chagas heart disease remains obscure. Here we used the antioxidant and cardioprotective agent resveratrol to test a novel, host-targeted strategy against established Chagas heart disease: promoting disease tolerance by tuning heart physiology to a healthy pattern. As our data reveal, treatment with resveratrol greatly improves electrical and contractile activities in Chagas heart disease, while activating AMPK pathway, reducing parasite burden and oxidative stress. The general picture that emerges from this study is that despite all tissue damage, there is a relevant physiological dysfunction in established Chagas heart disease which can be reversed to improve cardiac function, opening a new perspective for Chagas disease therapy.

## Results

### Resveratrol reverses most aspects of functional Chagas heart disease in mice

Murine models of Chagas disease do not usually present an indeterminate, silent stage, as happens in humans, and rather undergo a smooth transition from acute to chronic stage. We have chosen a model of infection with the Colombian strain of *T*. *cruzi*, which has been isolated from a patient in Colombia [27] and has been used in many functional heart studies in the last 25 years [16, 28, 29]. At 60 dpi, BALB/c infected with Colombian *T*. *cruzi* strain often present sinoatrial block, atrial abnormalities (atrial enlargement/ interatrial block), second-degree atrioventricular block, sinus arrhythmia, bradycardia, prolonged QTc interval, right ventricle dilation and moderately decreased left ventricular ejection fraction (LVEF), and mostly resembles the B1-B2 (NYHA class II) of human CCC [22, 30].

To test whether resveratrol is effective against established Chagas disease, we infected the highly susceptible BALB/c mice with the type I Colombian strain of *T*. *cruzi* and performed individual electrocardiography (ECG) and echocardiography studies before starting the treatment with resveratrol at 15 mg/Kg i.p. (at 60 days post-infection) and after treatment cycle was complete, at 90 days post-infection (dpi). Intraperitoneal route was chosen in order to increase the bioavailability of resveratrol [31]. In side studies, we tested 5 mg/Kg without success, while treatment with 10 mg/Kg presented promising results.

**Fig 1:**
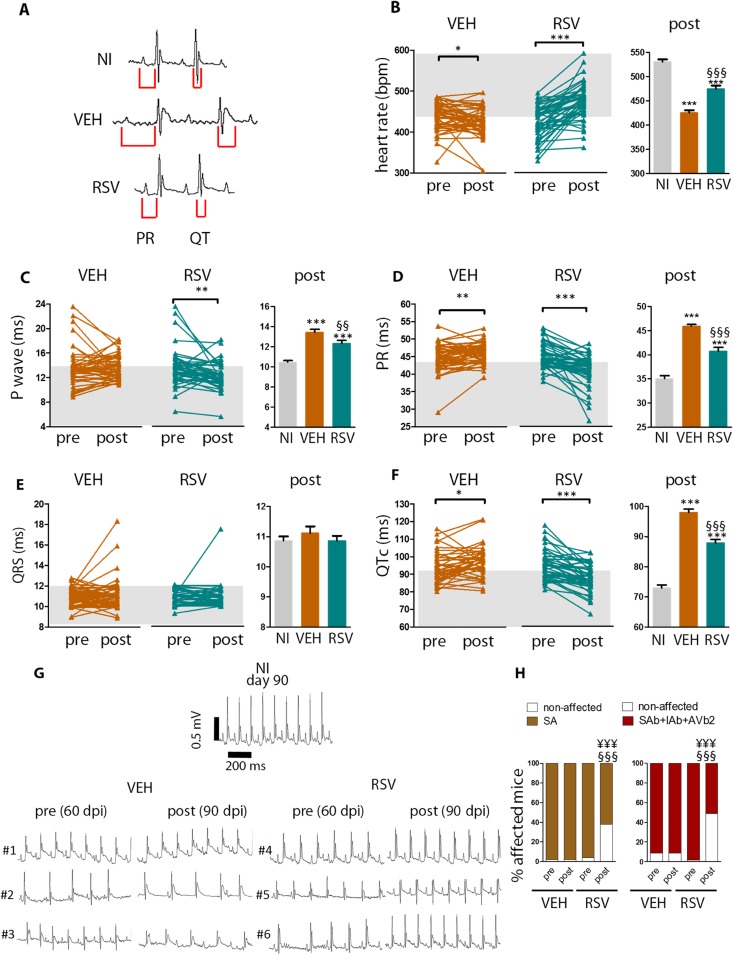
Resveratrol reverses ECG abnormalities in mice chronically infected with *T*. *cruzi*. Mice were individually identified, infected with Colombian-strain and treated with either resveratrol (RSV) or vehicle (VEH) from 60–90 dpi. Non-infected (NI) mice were kept as controls. (A), Representative tracings for each group at 90 dpi. (B-F), Left: evolution of ECG intervals for each individual mouse during the 60–90 dpi period. P-range is shown for paired comparisons. The gray shade represents the range of values for NI. Right: the ECG intervals at 90 dpi. P-range is shown for the unpaired comparisons. (G)**,** Representative ECG tracings for 3 individual mice per group before and after treatment. Note that atrial and atrioventricular conduction disorders (intra-atrial/ interatrial block, AIb, #1,#4), sinoatrial block (SAb, #2,#5), and second-degree atrioventricular block (AVb2, #3,#6), subsided in response to resveratrol, but not in response to vehicle. (H), incidence of sinus arrhythmia (SA, left) and atrial/ atrioventricular conduction disorders (right): sinoatrial block (SAb), intra-atrial/ interatrial block (IAb), and second-degree atrioventricular block (AVb2). Mice were pooled from six independent experiments (NI n = 46; VEH n = 49; RSV n = 47). Error bars indicate mean±SEM. *, different from NI; §, different from VEH. P range: *, § P≤0.05, ** §§, P<0.01, *** §§§ ¥¥¥, P<0.005

At 90 dpi, infected mice treated with resveratrol (RSV group) had faster heart rate and shorter P wave duration, PR, and QT intervals when compared to infected mice treated with vehicle (VEH group, Fig 1A, right graphs in Fig 1B, 1C, 1D and 1F). Non-infected controls (NI) are provided for comparison. Treatment with resveratrol shortened P wave duration, PR and QTc intervals, and increased individual heart rates during the interval from 60–90 dpi (left graphs in Fig 1B, 1C, 1D and 1F). On the other hand, vehicle did not significantly change P wave duration (left graphs in Fig 1C) and did not stop the progression towards even longer PR and QTc intervals and increasing bradycardia (left graphs in Fig 1D, 1F and 1B). ECG intervals did not differ between infected non-treated and infected vehicle-treated mice (S1A Fig). Similar beneficial results were obtained with peroral administration of a higher dose of resveratrol (S2 Fig, 40 mg/Kg).

Fig 1G displays representative ECG tracings for a non-infected control and 3 infected individuals before and after treatment with vehicle (#1–3) or resveratrol (#4–6). In the VEH group, incidence of sinus arrhythmia remained unchanged after treatment (Fig 1H). In contrast, treatment with resveratrol led to a 35% decrease in the percentage of sinus arrhythmia-affected mice (16 SA-free animals out of 45 previously affected). Treatment with resveratrol also produced a 49% decrease in the percentage of infected mice affected by atrial and atrioventricular conduction disorders (sinoatrial block, intra-atrial/ interatrial block, or second-degree atrioventricular block): 23 free animals out of 47 previously affected, while infected vehicle-treated mice had persistent abnormalities (Fig 1H). At 90 dpi (post-treatment), the incidence of conduction disorders was far greater among VEH than among RSV animals. Among RSV, 15/47 (31%) mice presented normal ECG profiles after treatment, while among VEH 48/48 (100%, p<0.0001) presented sinus arrhythmia, atrial and/ or atrioventricular conduction disorders.

Resveratrol instantly reversed ischemia-reperfusion arrhythmias ex vivo [6]. However, in our model, short-term treatment (20 h) did not present benefits on heart electrical function (S3A Fig). Treatment prorogation until 120 dpi maintained the benefits of resveratrol over vehicle on the heart electrical cycle (S3B Fig).

**Fig 2:**
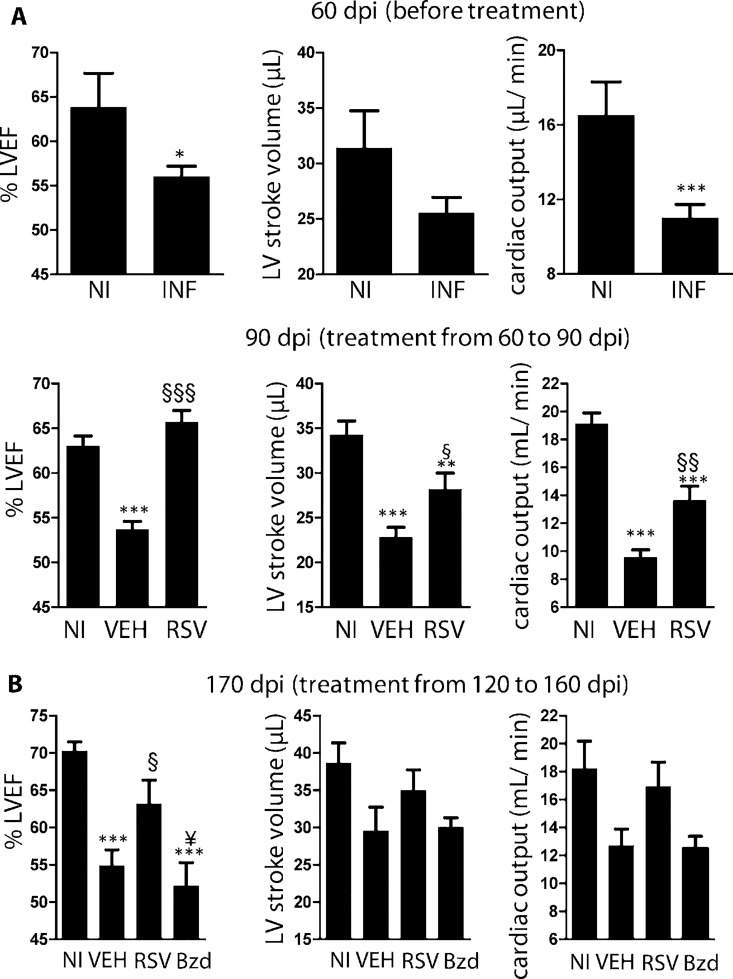
Resveratrol reverses heart-pumping dysfunction in mice chronically infected with *T*. *cruzi*. (A) *Upper panel*: echocardiography comparing heart function of non-infected (NI) and infected (INF) mice at 60 dpi (before treatment). Left ventricle ejection fraction (LVEF), left ventricle stroke volume and cardiac output. NI (n = 4), INF (n = 14), pooled from two independent experiments. *Lower panel*: at 90 dpi (after treatment): similar heart function parameters are shown for infected mice treated with vehicle (VEH, n = 20), resveratrol (RSV, n = 20) or for non-infected mice (NI, n = 23), pooled from three independent experiments. (B) Similar parameters shown for mice treated from 120 to 160 dpi, at 170 dpi. NI (n = 5), VEH (n = 5), RSV (n = 7), Bzd (n = 6). *, different from NI; §, different from VEH, ¥, different from resveratrol. P range: * § ¥, P≤0.05, ** §§, P<0.01, *** §§§, P<0.005

We used echocardiography to assess heart pumping efficiency. In previous studies, B-mode images were found to be more adequate to assess the geometry of chagasic hearts [32]. Before treatment (60 dpi), infected mice (INF) presented decreased left ventricle ejection fraction (LVEF), stroke volume, and cardiac output (Fig 2A), as well as dilated RV when compared with NI (S4A Fig). After treatment (90 dpi), VEH presented decreased LVEF on average, while RSV presented normal values (Fig 2A). Stroke volume and cardiac output were significantly improved in RSV compared to VEH animals.

Heart geometry changed in response to treatment. At 90 dpi, RV area was increased in VEH compared to NI mice, but treatment with resveratrol reversed RV dilation (S4A Fig). A transient decrease in LV area was found in all infected groups compared with NI mice at 90 dpi, similar to other models [33, 34], an effect no longer present at 120 dpi (S4 Fig). VEH did not significantly differ from non-treated infected mice concerning heart geometry (S1C Fig).

Even when mice were treated late after infection (120 dpi) with resveratrol for 40 days, they reacted positively to treatment, regaining a normal LVEF (Fig 2B) and presenting reduced right ventricle dilation at 170 dpi (S4B Fig). On the other hand, treatment with the trypanocidal drug benznidazole for 40 days starting at 120 dpi was not capable of improving LVEF (Fig 2B) or reducing right ventricle dilation (S4B Fig). Although treatment with resveratrol starting at 120 dpi failed to reduce P wave duration and QRS interval (which was prolonged at 170 dpi), it also promoted normal heart rates, decreased PR and QTc intervals (S5 Fig). Together, these results indicate that even when started late, treatment with resveratrol can still be beneficial to heart function.

Altogether, these results indicate that treatment with resveratrol has a profound beneficial effect on the cardiac function of mice with established Chagas disease, being able to partially reverse both contractile and electrical dysfunctions.

### Resveratrol does not reduce heart inflammatory infiltrates or vascularization

An inflammatory response characterized by leukocyte infiltration and tissue remodeling constitutes an important feature of CCC. Resveratrol did not significantly alter the number of invading inflammatory cells infiltrating the heart (S6A Fig), heart vascularization (S6B Fig), or collagen content (measured by either 2^nd^ harmonic or tricolor Masson,S6C and S6D Fig). A slight trend towards decreased number of infiltrating cells was found in RSV mice (S6A Fig, P = 0.30, 20 heart sections analyzed from each of 10–13 mice per group).

### Resveratrol activates the AMPK pathway, reduces parasite burden and heart oxidative stress

**Fig 3:**
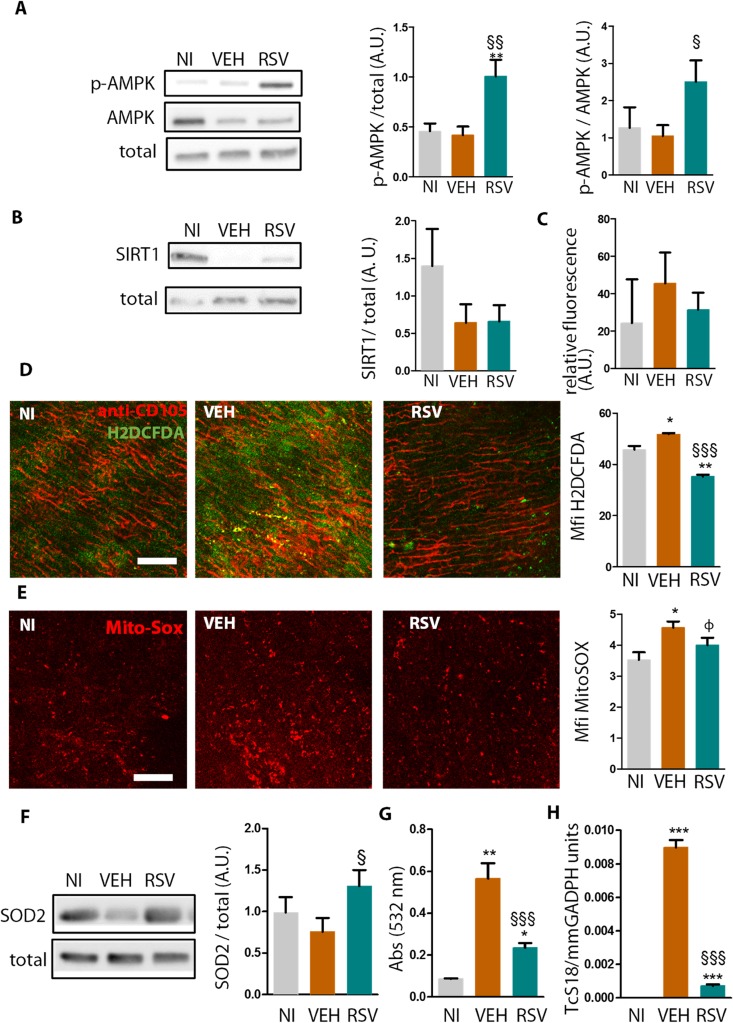
Resveratrol activates AMPK phosphorylation, reduces oxidative stress and parasite burden in the hearts of chronically infected mice. Mice were infected with Colombian-strain and treated with either resveratrol (RSV) or vehicle (VEH) from 60–90 dpi. (A), Ventricle extracts were probed at 90 dpi with antibodies against constitutive proteins (total), AMPK and p-AMPK. Bars show pAMPK/total or pAMPK/AMPK, sum of three independent experiments (NI n = 9, VEH n = 8, RSV n = 9 hearts/ group). A.U. = arbitrary units. (B), Similar to A, SIRT1/total, sum of three independent experiments, n = 9 hearts per group. (C), SIRT1 de-acetylation activity in ventricle extracts (n = 5). (D) Mean fluorescence intensity (MFI) for H_2_DCFDA oxidation (green) in fresh heart explants. The probe was injected i.v. in live mice along with labeled anti-CD105 (red) and analyzed by confocal microscopy, n = 3 hearts per group. Scale bar = 100 μm. (E) Similar to D, mito-Sox oxidation (red).NI and RSV (n = 4), VEH (n = 5) hearts/ group. (F) Similar to A, SOD2/total, n = 12 hearts/ group. (G) Lipid peroxidation assessed by TBARs in individual plasma samples. NI (n = 4), VEH (n = 6), and RSV (n = 10) hearts per group. (H) Relative amount of parasites (TcS18) per host DNA (GADPH) in heart tissue. Results represent mean±SEM for NI n = 6, VEH n = 8, RSV n = 12 hearts/ group. *, different from NI; §, different from VEH. P range: φ, P = 0.06; * §, P≤0.05, ** §§, P<0.01, *** §§§, P<0.005

Because resveratrol activates AMPK activation to restore cardiac function in other models [35, 36], we assessed AMPK phosphorylation (Thr 172) in the ventricles of VEH versus RSV animals at 90 dpi. Treatment with resveratrol promoted a significant increase in phosphorylated AMPK (normalized to the total protein levels) compared to the VEH and NI groups (Fig 3A), resulting in an increased p-AMPK/AMPK relation.

SIRT1 relative expression (Fig 3B) and de-acetylase activity (Fig 3C) were not significantly different among groups. These results suggest a SIRT1-independent effect of resveratrol, and in fact treatment of infected mice with resveratrol plus EX527, an specific SIRT1 inhibitor used as previously described [37], was not able to reverse the significant benefits of resveratrol on ECG abnormalities (S7 Fig). Together, these data support the notion that AMPK, but not SIRT1, is activated in response to resveratrol in CCC.

*T*. *cruzi* infection induces ROS production by cardiomyocytes [14], a phenomenon that may cause cardiomyocyte death, or may act physiologically to produce electrical and pumping dysfunction. We assessed extravascular ROS production using in vivo CD105 (endothelial marker)/DCDFA labeling at 90 dpi. Extravascular ROS was increased in VEH group when compared to NI, while RSV had ROS levels below those found in NI mice (Fig 3D). In vivo MitoSOX staining was significantly greater in VEH compared to NI mice (Fig 3E). We observed a greater staining in VEH compared to RSV hearts (P = 0.06). RSV mice had increased heart expression of the mitochondrial enzyme SOD2, an AMPK-controlled enzyme [38], compared with VEH mice (Fig 3F). Mitochondrial oxidative stress has been previously suggested as a possible causative factor for chagasic heart dysfunction [39, 40] Treatment with resveratrol, in comparison with vehicle, greatly reduced lipid peroxidation in plasma, as assessed by thiobarbituric acid reactive substances (TBARs) (Fig 3G). A sharp decrease in heart tissue parasitism was detected by quantitative PCR in RSV when compared with VEH mice at 90 dpi (Fig 3H).

We assessed the expression of some proteins controlled by AMPK/SIRT1. Contrary to our expectations, there was an increase in p-ACC and GLUT4 in infected mice and resveratrol promoted a decrease towards non-infected levels (S8 Fig), an effect probably due to resveratrol’s remarkable effect of reducing parasite burden. We did not find any significant differences in PGC1α expression or cardiac ATP levels.

These results show that resveratrol activates the AMPK pathway and decreases parasite burden, together with reducing ROS production and lipid peroxidation.

### Metformin and tempol decrease lipid peroxidation and improve heart function in CCC, but do not reduce parasite burden

Because resveratrol activated AMPK phosphorylation and reduced ROS in our Chagas heart disease model, we tested whether activating AMPK or reducing ROS could mimic the beneficial effects of resveratrol on heart function.

Metformin (Met) is an AMPK activator and a cardioprotective drug [41, 42] and has indirect antioxidant activity, increasing the expression of antioxidant enzymes such as SOD2 [38]. Tempol (Tmp) is a SOD-mimetic drug that efficiently neutralizes ROS [43]. These drugs were administered daily by gavage. We also performed the usual i.p. treatment with RSV and respective VEH to allow a comparison of heart effects between RSV, Met, and Tmp.

**Fig 4:**
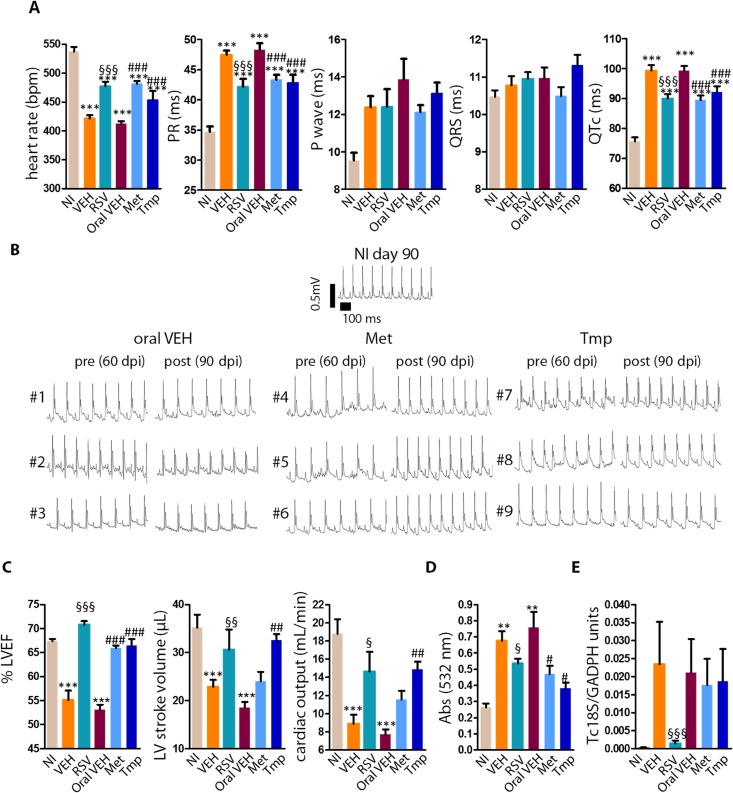
Effects of metformin and tempol on heart function, lipid peroxidation and parasite burden of chronically infected mice. Infected mice were treated with peroral metformin (Met), tempol (Tmp), or oral vehicle (VEH) from 60–90 dpi. Infected mice treated i.p. with resveratrol (RSV) or vehicle (VEH) were kept as controls, as well as non-infected mice. Heart function, oxidative damage and parasite burden were assessed at 90 dpi. (A) heart rate, PR interval, P duration, QRS duration, QTc interval. Mice per group: NI (n = 11), VEH (n = 7), Met (n = 9), Tmp (n = 9). Met are representative of 2 similar experiments. (B) Pre- and post-treatment ECG tracings. Picture illustrate the reversion of abnormalities in Met and Tmp, but not in VEH group, in three individual mice from each group (#1–9). (C) Left ventricle ejection fraction (LVEF), stroke volume, right ventricle area (RV area); left ventricle area (LV area). Mice per group: NI (n = 10), VEH (n = 8), Met (n = 9), Tmp (n = 8). (D), Lipid peroxidation (TBARs) in heart extracts. Mice per group: NI (n = 3), VEH (n = 3), Met (n = 7–9), Tmp (n = 8).(E) Relative amount of parasites (TcS18) per host DNA (GADPH) in individual heart tissue. Mice per group: NI (n = 6), VEH (n = 5), Met (n = 6), Tmp (n = 5). *, different from NI; §, different from VEH, #, different from oral VEH. P range: * § #, P≤0.05, ** §§ ##, P<0.01, *** §§§ ###, P<0.005

Mice treated with Met or Tmp had decreased PR and QTc intervals and increased heart rates compared to peroral VEH (Fig 4A). These results were similar to that obtained by treatment with RSV. Pre- and post-treatment profiles of individual mice are illustrated in Fig 4B (#1–9). Met decreased the percentage of arrhythmias and conduction disorders among infected mice: while 7/7 (100%) animals treated with vehicle presented sinus arrhythmia, sinoatrial block, intra-atrial/ interatrial block, and/ or second-degree atrioventricular block at 90 dpi, only 4/9 (44%, VEH x Met P = 0.03) among those treated with Met were positive.

Treatment with either Met or Tmp also restored a normal LVEF (Fig 4C) and Tmp significantly increased stroke volume and cardiac output when compared to peroral VEH. The results obtained with Tmp were similar to that obtained by treatment with RSV. Tmp also significantly reduced right ventricle dilation (S9 Fig). Importantly, Met and Tmp reduced lipid peroxidation of heart samples (Fig 4D), but did not alter heart parasite burden (Fig 4E).

Though mimicking the results obtained with RSV using Met or Tmp does not actually demonstrate that RSV acted in the same way the other drugs did, it indicates that AMPK activation and SOD mimetic activity can be exploited as therapeutic strategies in Chagas heart disease. Taken together, these results suggest that reducing ROS is sufficient to improve heart function in CCC, while decreasing parasite burden is not required to improve heart function.

## Discussion

**Fig 5:**
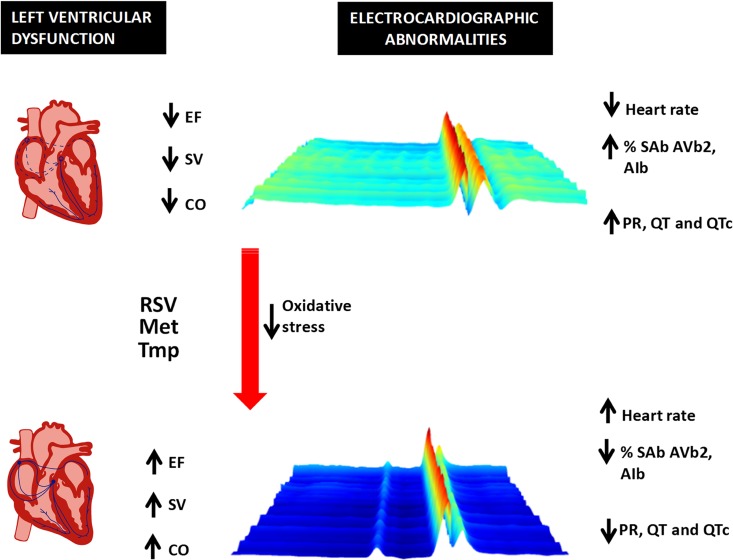
Diagrammatic illustration of the effects of resveratrol on established Chagas heart disease. Treatment with resveratrol, an AMPK activator (metformin), or a ROS scavenger (tempol) were capable of ameliorating cardiac function in Chagas disease. The drugs reduced the incidence of sinus arrhythmia (SA), atrial abnormalities, intra-atrial and interatrial block (AIb), sinoatrial block (SAb), second-degree atrioventricular block (AVb2), reduced the QT and PR intervals, increased the heart rate, increased ejection fraction (EF), stroke volume (SV), and cardiac output (CO). The 3D ECG tracing was chosen from a mouse before and after treatment with resveratrol.

The several attempts to treat CCC with trypanocidal drugs have produced inconsistent results, despite reductions in parasite load. Infected mice have been studied pre- and post-treatment with benznidazole [18]: treatment eliminated the parasite and prevented to a small extent the prolongation of the PR interval over time, but by the end of the study, benznidazole-treated mice offered no improvements over controls. A study of infected rats treated with benznidazole found no improvement of heart function analyzed by catheterism [15]. In human CCC, an attempt to reduce parasite load with benznidazole cured heart disease in some cases [19], but not in another study [20]. The recent results of the BENEFIT trial of benznidazole at the chronic phase show that despite greatly reducing parasite burden, it does not affect cardiac deterioration [21]. These results reinforce the notion that heart pathology and parasite burden have a loose association at the chronic stage and discourage the trypanocidal strategy against disease. Here, we show that resveratrol improves heart function (a diagrammatic illustration of its heart function effects in CCC is shown in Fig 5) and reduces heart parasite burden. Nevertheless, we believe that instead of acting primarily by reducing parasite burden to improve heart function, resveratrol acted as an antioxidant. Different from resveratrol, trypanocidal drug benznidazole failed to restore heart pumping function. Moreover, both metformin (AMPK activator) and tempol (SOD-mimetic) improved heart function and decreased lipid peroxidation, but did not change parasite burden. These findings support raising disease tolerance as an effective strategy against CCC, as long as the parasite burden is kept low in order to avoid a rebound.

We have previously shown that oxidative stress fuels acute *T*. *cruzi* infection in mice [24], promoting parasitism in heart and macrophages. In that previous study, resveratrol was able to reduce acute parasitemia and macrophage parasite burden. Here we show that at the chronic phase of infection, resveratrol, AMPK-activator metformin and SOD-mimetic tempol reduce lipid peroxidation, a measure of oxidative stress, but only resveratrol reduces heart parasite burden. We believe that at this stage of infection, oxidative stress caused by respiratory burst in macrophages is no longer a significant factor promoting *T*. *cruzi* infection in mice. In fact, resveratrol was recently demonstrated to have a direct trypanocidal effect [44], and therefore any of its host-dependent antioxidant effects can be dispensed with to explain the reduction of heart parasite burden it promoted.

Exercise and a healthy diet remain the cornerstones of prevention and treatment of heart disease. Some drugs mimic the effects of diet and exercise by triggering signals that resemble those seen with reduced ATP levels and activating the phosphorylation of AMPK. By doing so, these drugs protect the heart [3]. Here, we show that treatment with one of these drugs, resveratrol, also promoted phosphorylation of AMPK in the hearts of infected mice, suggesting that AMPK activation is likely to be one of the cardioprotective mechanisms in this case [3]. Consistently, metformin, an indirect AMPK activator [45], mimicked most of the effects of resveratrol on heart electrical function and its effects on ejection fraction. SOD2, an AMPK-controlled mitochondrial antioxidant enzyme [38], was found to be significantly increased by treatment with resveratrol and general SOD-mimetic tempol was able to mimic resveratrol’s effects on heart function. These results indicate that the AMPK-pathway and its effects on oxidative stress are a likely target to resveratrol, though not the only one. Future studies are required to define the exact mechanism by which resveratrol promotes its beneficial effects in Chaga’s Disease. Resveratrol is a multitarget drug that besides activating AMPK pathway [4], interacts directly with 20 proteins [46]: it activates Nrf2 gene [47], has a direct antioxidant effect and is a PDE4 inhibitor [48], and it is likely that its heart effects in chronic Chagas disease depend on several of these mechanisms.

Our results showed that in infected, vehicle treated mice, there was an increase in phosphorylation of ACC that did not depend on AMPK activation. Although in normal heart resveratrol promotes an increase in phosphorylation of ACC [36], in this case, it worked to prevent the increase in p-ACC promoted by infection. A similar situation seemed to happen to GLUT4, a gene controlled by AMPK activation and similarly increased in infected vehicle and resveratrol-treated mice. We do not know the reasons for these surprising findings, but we believe infection activates ACC phosphorylation through a pathway other than AMPK while resveratrol reduces the activity of this pathway by reducing the parasite burden. We also did not expect the effects of resveratrol to be independent of SIRT1 activation, but AMPK is known to be activated by high doses of resveratrol independently of SIRT1 [49].

The association between ROS production and heart disease is well known. Here we show that antioxidants resveratrol and tempol reduced oxidative stress and improved heart function in established Chagas heart disease. Neither AMPK activators nor antioxidants have previously been tried as a therapy for established Chagas heart disease. In a previous study, antioxidant phenyl-tert-butyl-nitrone was administered before infection (starting at day 0) until the chronic phase, preventing the establishment of functional Chagas heart disease in rats [15]. Although that study bears similarities to ours, indicating a role for ROS in heart functional damage, no treatment has been shown to reverse established Chagas heart disease until now. Because trypanocidal therapy is only effective during acute stage but diagnosis usually occurs during the chronic disease stage, strategies to reverse established heart disease are welcome, while new strategies to prevent its progression have few applications. Our data indicate that reduction of oxidative stress likely represents a viable therapeutic strategy in CCC, for which there are currently no effective therapies. The mechanisms by which ROS causes cardiac dysfunction in Chagas heart disease are now unclear.

The improvement in cardiac function observed in chronically infected mice after treatment with resveratrol challenges the current paradigm about Chagas disease pathogenesis. It is currently believed that the cumulative tissue damage caused by infection and inflammation breaks the functional structure of the heart, and can only be reversed by replacing cardiac tissue with stem cells. Previous reports show that some combinations of *T*. *cruzi* strains and mice fail to alter ECG recordings, while presenting as much inflammatory infiltrates/ tissue disorganization as combinations that do alter ECG recordings. These findings indicate that functional disease does not easily correlate with tissue damage [50]. We showed here that reversal of heart dysfunction was not associated with decreased inflammatory infiltrates, while others found that antioxidant-induced prevention of heart dysfunction did not alter inflammatory infiltrates [15]. We hypothesize that there are two overlapping heart dysfunctions in CCC: one that is merely physiological and easily reversible and another that is structural, involves inflammatory infiltrates, cell death and extensive tissue remodeling. Oxidative stress probably underlies both dysfunctions. Based on our data, we speculate that heart cells react to chronic infection/ inflammation with changes in physiology that lead to high ROS production. The heart disease that follows is somewhat similar to that found in other ROS-related cardiomyopathies. Our results show that despite all the tissue damage found in established Chagas heart disease, the physiological impairment affecting the heart is still reversible and the heart function can be significantly improved, offering a new therapeutic opportunity to millions of patients suffering from Chronic chagasic cardiomyopathy worldwide.

Our study has some limitations and in order to overcome them and translate our study into a clinical trial, we still plan to perform a full study of cardiac function after resveratrol treatment, approaching the following questions: (1) oral treatment with extended bioavailability, using co-administration of glucuronidation inhibitor piperine, [51]; (2) treatment interruption and analysis after various intervals; (3) extended treatment (until 200 dpi); (4) treatment of chronic chagasic mice infected by other *T*. *cruzi* strains.

Our results show resveratrol reverses important aspects of this heart dysfunction: shortens QTc, an independent risk factor for sudden cardiac arrest; reverses atrial and atrioventricular conduction disorders, risk factors for cardiovascular mortality; and restores ejection fraction, a major contributor to morbidity [22, 30]. In addition, resveratrol greatly decreases heart parasite burden, reducing concerns of infection rebound. As resveratrol is considered a food supplement to most health agencies, such as US Food and Drug Administration, we believe that resveratrol is a suitable candidate to human trials in a very near future, targeting B1-B2 clinical stage of CCC.

## Materials and Methods

The complete procedures for electrocardiography, histopathological studies, SIRT1 deacetylase activity, ATP dosage, lipid peroxidation, quantitative Polymerase Chain Reaction (qPCR) for parasite detection, western blot, ex-vivo heart confocal microscopy for ROS and vessels, and second-harmonic generation for collagen detection are described on an online appendix.

### Ethics statement

This study was carried out in strict accordance with the recommendations of the Guide for the Care and Use of Laboratory Animals of the Brazilian National Council of Animal Experimentation (http://www.cobea.org.br/) and Federal Law 11.794 (October 8, 2008). The institutional Committee for Animal Ethics of UFRJ (CEUA, Licenses IMPPG029 e IMPPG032.) and Fiocruz (Licenses 004/09 and LW10-14) approved all the procedures used in this study.

### Mice

Female or male BALB/c mice (5–7 weeks of age) obtained from the animal facilities (CECAL) of the Oswaldo Cruz Foundation (Fiocruz, Rio de Janeiro, Brazil) and Universidade de São Paulo, Brazil were kept in a sterile environment under standard conditions (temperature and relative humidity of approximately 22 ± 2°C and 55 ± 10%, respectively) and received food and water *ad libitum*. Mice were individually identified by ear tags.

### Infection and mice treatment

Mice were infected intraperitoneally (i.p.) with 10^2^ blood trypomastigote forms of the type I Colombian strain of *T*. *cruzi*. Treatments were performed daily for 30 days from the establishment of CCC (60 dpi) by i.p. injection of 15 mg.kg^-1^ trans-resveratrol (Sigma, 10% ethanol/PBS), vehicle (10% ethanol/PBS), 5 mg.Kg^-1^ EX527 (0.1% DMSO, Sigma), or peroral administration of 40 mg.Kg^-1^ resveratrol (10%ethanol-PBS), 500 mg.kg^-1^ metformin (Merck, dissolved in water), 100 mg.kg^-1^ tempol (Sigma, dissolved in water), benznidazole (Rochagan, 25 mg/Kg, dissolved in water) and vehicle (water or 10%ethanol-PBS).

### Electrocardiography (ECG)

Mice were sedated with diazepam (10 mg/kg) and transducers were placed subcutaneously (DII derivation). The traces were recorded for 2 minutes using the digital Power Lab 2/20 or Power Lab 4/35 Systems connected to a bio-amplifier (PanLab Instruments, Spain). The filters were standardized to 0.1-100Hz and the traces were analyzed with Scope for Windows (V3.6.10, PanLab instruments). Further details are provided in Online Methods. The assessment of P wave duration and incidence of conduction disorders required large numbers of mice to provide a valid statistical analysis.

### Transthoracic echocardiography (echo)

Echo was performed under deep isoflurane anesthesia (2% in oxygen). Mice were trichotomized in the precordial region using depilatory cream and examined under a 30 Mhz transducer with a Vevo 770 Ultrasound apparatus (Visual Sonics, Canada). The left ventricle ejection fraction (LVEF) was calculated using Simpson’s method, chosen because of its fit with CD heart geometry and because it is commonly used to assess CD patients. The area of the left and right ventricles during diastoles and systoles were obtained in B mode using a short axis view at the level of the papillary muscles.

### Statistical analyses

Most of the comparisons between means ± SEMs were made using unpaired Student’s t tests (two groups) or one-way ANOVA with Newman-Keuls post-test (multiple groups), except for pre versus post analyses, in which we used paired Student’s t tests. The comparison between the incidences of arrhythmia across groups was calculated using Fisher’s exact t test. Differences with a p-value <0.05 were considered significant and significant p-values are shown in the figures next to the compared groups.

## Supporting Information

S1 Fig Cardiac function of infected non-treated and infected vehicle-treated mice do not differ. (A), ECG intervals at 90 dpi for non-infected (NI), infected non-treated (NT), or (VEH)-treated mice. (B) Left ventricle ejection fraction (LVEF), left ventricle stroke volume, cardiac output; (C) right ventricle area (RV area), left ventricle area (LV area). NI (n = 4), NT (n = 5), VEH (n = 6), representative of two independent experiments. Error bars indicate mean±SEM. *, different from NI. P range: *, P≤0.05, **, P<0.01, ***, P<0.005.
(TIF)

S2 Fig Electrical cardiac function of infected mice treated by peroral or intraperitoneal route with vehicle or resveratrol. Mice were treated from 60–90 dpi with resveratrol or vehicle through either intraperitoneal (15 mg/Kg) or peroral (40 mg/Kg) routes. ECG intervals at 90 dpi for noninfected (NI), infected peroral vehicle-treated (VEH p.o.), infected peroral resveratrol-treated (RSV p.o.), infected intraperitoneal vehicle-treated (VEH i.p.), infected intraperitoneal resveratrol-treated mice (RSV i.p.) NI (n = 9), VEH p.o. (n = 8), RSV p.o. (n = 4), VEH i.p. (n = 4), RSV i.p. (n = 5). Error bars indicate mean±SEM. *, different from NI; §, different from respective VEH. P range: * §, P≤0.05, ** §§, P<0.01, ***, P<0.001
(TIF)

S3 Fig Resveratrol affects ECG recordings after a latency period, and once its effects are established, they can be maintained for long periods. (A), ECG intervals before and 20 h after treatment of infected mice (60 dpi) with vehicle (VEH) or resveratrol (RSV). Mice per group: NI (n = 5), VEH (n = 10), and RSV (n = 10). (B), ECG intervals before (60 dpi) and throughout treatment (90 dpi, 120 dpi) of infected mice with vehicle or resveratrol. Mice per group: NI (n = 4–10), VEH (n = 4–10), RSV (n = 8–10). A physiological zero was adopted on the Y axis (lowest value found by us in mice). The Pvalue was calculated using ANOVA to compare groups at 120 dpi. Error bars indicate mean±SEM. *, different from NI; §, different from VEH. P range: §, P≤0.05, **, P<0.01, ***, P<0.005
(TIF)

S4 Fig Resveratrol affects heart geometry in infected mice. (A) *Upper panel*: echocardiography comparing heart geometry between non-infected (NI) and infected (INF) mice at 60 dpi (before treatment). Left ventricle area, right ventricle area and left ventricle diastolic volume. NI (n = 4), INF (n = 14), pooled from two independent experiments. *Lower panel*: at 90 dpi (after treatment): similar heart geometry parameters are shown for infected mice treated with vehicle (VEH, n = 20) or resveratrol (RSV, n = 20) or non-infected mice (NI, n = 23), pooled from three independent experiments. (B) Similar parameters shown for mice treated from 120 to 160 dpi, at 170 dpi. NI (n = 5), VEH (n = 5), RSV (n = 7), Bzd (n = 6). *, different from NI; §, different from VEH. P range: * §, P≤0.05, *** §§§, P<0.005
(TIF)

S5 Fig Resveratrol improves heart electrical cycle late after infection is established. Infected mice were treated with either resveratrol (RSV) or vehicle (VEH) from 120–160 dpi and their heart electrical function was assessed by ECG at 170 dpi. NI (n = 5), VEH (n = 5), RSV (n = 7). Error bars indicate mean±SEM. *, different from NI; §, different from VEH. P range: *, § P≤0.05, ** §§, P<0.01.
(TIF)

S6 Fig Resveratrol has little effect on the histological structure of the heart in CCC. Heart histology was assessed 30 days after treatment of infected mice with either vehicle or resveratrol (at 90 dpi). (A) H&E heart slides and graph showing inflammatory infiltrates in the hearts of infected vehicle (VEH)- or resveratrol (RSV)-treated mice. No differences were found among ventricles. Hearts per group: NI (n = 8), VEH (n = 13), and RSV (n = 10), pooled from 2 independent experiments. (B) Confocal microscopy of fresh heart explants from mice injected in vivo with labeled anti-CD105 to reveal blood vessels, n = 3 hearts / group. (C) 2^nd^ harmonic imaging microscopy of fixed heart explants to reveal collagen (green) and structural myocardium proteins (red). Interstitial collagen was estimated from these images. Hearts per group: NI (n = 3), VEH (n = 5) and RSV (n = 5). (D) 3-color Masson was used to reveal interstitial collagen in left ventricle. No significant differences were found among VEH and RSV ventricles. n = 9 hearts / group. Error bars indicate mean±SEM. *, different from NI. P range: **, P<0.01, ***, P<0.005.
(TIF)

S7 Fig SIRT1-inhibitor EX527 does not prevent resveratrol effects on heart electrical cycle. Infected mice were treated with either resveratrol (RSV) or vehicle (VEH) from 60–90 dpi and their heart electrical function was assessed by ECG at 90 dpi. NI (n = 5), VEH (n = 5), RSV (n = 7). Error bars indicate mean±SEM. NI n = 11, VEH n = 10, RSV n = 13, EX527+RSV n = 9. *, different from NI; §, different from VEH. P range: *, § P≤0.05, ** §§, P<0.01, ***, P<0.005.
(TIF)

S8 Fig Resveratrol promotes a non-infected pattern of key proteins in heart metabolism. (A) Ventricle extracts were probed 30 days after treatment of infected mice (with either vehicle or resveratrol) with antibodies against ACC (ACC2 is the top band), p-ACC, and constitutive control (total). Results are shown for ACC2. Sum of three independent experiments (n = 9 hearts per group). (B) Similar to (A), individual ventricle extracts were probed for PGC1α (top band). Hearts per group: NI n = 5, VEH n = 6, RSV n = 6 (sum of two independent experiments) or (C) GLUT4 (n = 3 hearts per group). (D) ATP amounts in ventricle extracts. Pooled from two independent experiments (NI n = 5, VEH n = 6, RSV n = 5 hearts per group). Error bars indicate mean±SEM. *, different from NI; §, different from VEH. P range: *, § P≤0.05.
(TIF)

S9 Fig SOD-mimetic tempol affects heart geometry in infected mice. Infected mice were treated with peroral metformin (Met), tempol (Tmp), or oral vehicle (VEH) from 60–90 dpi. Infected mice treated i.p. with resveratrol (RSV) or vehicle (VEH) were kept as controls, as well as non-infected mice. Left ventricle area, right ventricle area and left ventricle diastolic volume. Mice per group: NI (n = 11), VEH (n = 7), Met (n = 9), Tmp (n = 9). Met are representative of 2 similar experiments. *, different from NI; §, different from VEH, #, different from oral VEH. P range: *, P≤0.05, **, P<0.01, *** §§§ ###, P<0.005
(TIF)

S1 Text Online Methods. (DOCX)

## References

[1] Medzhitov R, Schneider DS, Soares MP. Disease tolerance as a defense strategy. Science. 2012;335(6071):936–41. PubMed Central PMCID: PMC3564547. 10.1126/science.1214935 22363001 PMC3564547

[2] Jamieson AM, Pasman L, Yu S, Gamradt P, Homer RJ, Decker T, et al Role of tissue protection in lethal respiratory viral-bacterial coinfection. Science. 2013;340(6137):1230–4. PubMed Central PMCID: PMC3933032. 10.1126/science.1233632 23618765 PMC3933032

[3] Kim TT, Dyck JR. Is AMPK the savior of the failing heart? Trends in endocrinology and metabolism: TEM. 2015;26(1):40–8. 10.1016/j.tem.2014.11.001 25439672

[4] Raj P, Louis XL, Thandapilly SJ, Movahed A, Zieroth S, Netticadan T. Potential of resveratrol in the treatment of heart failure. Life sciences. 2014;95(2):63–71. 10.1016/j.lfs.2013.12.011 24361400

[5] Li W, Wang YP, Gao L, Zhang PP, Zhou Q, Xu QF, et al Resveratrol protects rabbit ventricular myocytes against oxidative stress-induced arrhythmogenic activity and Ca2+ overload. Acta pharmacologica Sinica. 2013;34(9):1164–73. PubMed Central PMCID: PMC4003166. 10.1038/aps.2013.82 23912472 PMC4003166

[6] Chen WP, Su MJ, Hung LM. In vitro electrophysiological mechanisms for antiarrhythmic efficacy of resveratrol, a red wine antioxidant. European journal of pharmacology. 2007;554(2–3):196–204. 10.1016/j.ejphar.2006.10.016 17107672

[7] Zhang Y, Liu Y, Wang T, Li B, Li H, Wang Z, et al Resveratrol, a natural ingredient of grape skin: antiarrhythmic efficacy and ionic mechanisms. Biochemical and biophysical research communications. 2006;340(4):1192–9. 10.1016/j.bbrc.2005.12.124 16406237

[8] Baczko I, Liknes D, Yang W, Hamming KC, Searle G, Jaeger K, et al Characterization of a novel multifunctional resveratrol derivative for the treatment of atrial fibrillation. British journal of pharmacology. 2014;171(1):92–106. PubMed Central PMCID: PMC3874699. 10.1111/bph.12409 24102184 PMC3874699

[9] Xin P, Pan Y, Zhu W, Huang S, Wei M, Chen C. Favorable effects of resveratrol on sympathetic neural remodeling in rats following myocardial infarction. European journal of pharmacology. 2010;649(1–3):293–300. 10.1016/j.ejphar.2010.09.036 20869962

[10] Park EJ, Pezzuto JM. The pharmacology of resveratrol in animals and humans. Biochimica et biophysica acta. 2015;1852(6):1071–113. 10.1016/j.bbadis.2015.01.014 25652123

[11] Cunha-Neto E, Chevillard C. Chagas disease cardiomyopathy: immunopathology and genetics. Mediators of inflammation. 2014;2014:683230 PubMed Central PMCID: PMC4152981. 10.1155/2014/683230 25210230 PMC4152981

[12] Rassi A Jr., Rassi A, Marin-Neto JA. Chagas disease. Lancet. 2010;375(9723):1388–402. 10.1016/S0140-6736(10)60061-X 20399979

[13] Dhiman M, Garg NJ. NADPH oxidase inhibition ameliorates Trypanosoma cruzi-induced myocarditis during Chagas disease. The Journal of pathology. 2011;225(4):583–96. 10.1002/path.2975 21952987 PMC4378678

[14] Wen JJ, Garg NJ. Mitochondrial generation of reactive oxygen species is enhanced at the Q(o) site of the complex III in the myocardium of Trypanosoma cruzi-infected mice: beneficial effects of an antioxidant. Journal of bioenergetics and biomembranes. 2008;40(6):587–98. 10.1007/s10863-008-9184-4 19009337 PMC6427913

[15] Wen JJ, Gupta S, Guan Z, Dhiman M, Condon D, Lui C, et al Phenyl-alpha-tert-butyl-nitrone and benzonidazole treatment controlled the mitochondrial oxidative stress and evolution of cardiomyopathy in chronic chagasic Rats. Journal of the American College of Cardiology. 2010;55(22):2499–508. PubMed Central PMCID: PMC2887697. 10.1016/j.jacc.2010.02.030 20510218 PMC2887697

[16] Pereira IR, Vilar-Pereira G, Moreira OC, Ramos IP, Gibaldi D, Britto C, et al Pentoxifylline Reverses Chronic Experimental Chagasic Cardiomyopathy in Association with Repositioning of Abnormal CD8+ T-Cell Response. PLoS neglected tropical diseases. 2015;9(3):e0003659 10.1371/journal.pntd.0003659 25789471 PMC4366205

[17] Garcia S, Ramos CO, Senra JF, Vilas-Boas F, Rodrigues MM, Campos-de-Carvalho AC, et al Treatment with benznidazole during the chronic phase of experimental Chagas disease decreases cardiac alterations. Antimicrobial agents and chemotherapy. 2005;49(4):1521–8. PubMed Central PMCID: PMC1068607. 10.1128/AAC.49.4.1521-1528.2005 15793134 PMC1068607

[18] Zaidenberg A, Luong T, Lirussi D, Bleiz J, Del Buono MB, Quijano G, et al Treatment of experimental chronic chagas disease with trifluralin. Basic & clinical pharmacology & toxicology. 2006;98(4):351–6. 16623857 10.1111/j.1742-7843.2006.pto_253.x

[19] Viotti R, Vigliano C, Lococo B, Bertocchi G, Petti M, Alvarez MG, et al Long-term cardiac outcomes of treating chronic Chagas disease with benznidazole versus no treatment: a nonrandomized trial. Annals of internal medicine. 2006;144(10):724–34. 16702588 10.7326/0003-4819-144-10-200605160-00006

[20] Molina I, Gomez i Prat J, Salvador F, Trevino B, Sulleiro E, Serre N, et al Randomized trial of posaconazole and benznidazole for chronic Chagas disease. The New England journal of medicine. 2014;370(20):1899–908. 10.1056/NEJMoa1313122 24827034

[21] Morillo CA, Marin-Neto JA, Avezum A, Sosa-Estani S, Rassi A Jr., Rosas F, et al Randomized Trial of Benznidazole for Chronic Chagas Cardiomyopathy. The New England journal of medicine. 2015. 10.1056/NEJMoa1507574 26323937

[22] Healy C, Viles-Gonzalez JF, Saenz LC, Soto M, Ramirez JD, d'Avila A. Arrhythmias in Chagasic Cardiomyopathy. Card Electrophysiol Clin. 2015;7(2):251–68. 10.1016/j.ccep.2015.03.016 26002390

[23] de Carvalho AC, Carvalho AB, Goldenberg RC. Cell-based therapy in Chagas disease. Advances in parasitology. 2011;75:49–63. 10.1016/B978-0-12-385863-4.00003-4 21820551

[24] Paiva CN, Feijo DF, Dutra FF, Carneiro VC, Freitas GB, Alves LS, et al Oxidative stress fuels Trypanosoma cruzi infection in mice. The Journal of clinical investigation. 2012;122(7):2531–42. PubMed Central PMCID: PMC3386808. 10.1172/JCI58525 22728935 PMC3386808

[25] Paiva CN, Bozza MT. Are reactive oxygen species always detrimental to pathogens? Antioxidants & redox signaling. 2014;20(6):1000–37. PubMed Central PMCID: PMC3924804. 23992156 10.1089/ars.2013.5447 PMC3924804

[26] Kayama Y, Raaz U, Jagger A, Adam M, Schellinger IN, Sakamoto M, et al Diabetic Cardiovascular Disease Induced by Oxidative Stress. International journal of molecular sciences. 2015;16(10):25234–63. PubMed Central PMCID: PMCPMC4632800. 10.3390/ijms161025234 26512646 PMC4632800

[27] Federici EE, Abelmann WH, Neva FA. Chronic and Progressive Myocarditis and Myositis in C3h Mice Infected with Trypanosoma Cruzi. The American journal of tropical medicine and hygiene. 1964;13:272–80. 14125879 10.4269/ajtmh.1964.13.272

[28] Falasca CA, Grana DR, Mareso EA, Gomez E, Gili MM. Electrocardiographic changes in chronic Trypanosoma cruzi infected Cebus apella monkeys. Arquivos brasileiros de cardiologia. 1991;56(4):287–93. 1909524

[29] Rocha NN, Garcia S, Gimenez LE, Hernandez CC, Senra JF, Lima RS, et al Characterization of cardiopulmonary function and cardiac muscarinic and adrenergic receptor density adaptation in C57BL/6 mice with chronic Trypanosoma cruzi infection. Parasitology. 2006;133(Pt 6):729–37. 10.1017/S0031182006001193 16978452

[30] Ribeiro AL, Nunes MP, Teixeira MM, Rocha MO. Diagnosis and management of Chagas disease and cardiomyopathy. Nature reviews Cardiology. 2012;9(10):576–89. 10.1038/nrcardio.2012.109 22847166

[31] Amri A, Chaumeil JC, Sfar S, Charrueau C. Administration of resveratrol: What formulation solutions to bioavailability limitations? J Control Release. 2012;158(2):182–93. 10.1016/j.jconrel.2011.09.083 21978644

[32] Mello DB, Ramos IP, Mesquita FC, Brasil GV, Rocha NN, Takiya CM, et al Adipose Tissue-Derived Mesenchymal Stromal Cells Protect Mice Infected with Trypanosoma cruzi from Cardiac Damage through Modulation of Anti-parasite Immunity. PLoS neglected tropical diseases. 2015;9(8):e0003945 PubMed Central PMCID: PMCPMC4527728. 10.1371/journal.pntd.0003945 26248209 PMC4527728

[33] Mukherjee S, Machado FS, Huang H, Oz HS, Jelicks LA, Prado CM, et al Aspirin treatment of mice infected with Trypanosoma cruzi and implications for the pathogenesis of Chagas disease. PloS one. 2011;6(2):e16959 PubMed Central PMCID: PMC3039660. 10.1371/journal.pone.0016959 21347238 PMC3039660

[34] Jelicks LA, Shirani J, Wittner M, Chandra M, Weiss LM, Factor SM, et al Application of cardiac gated magnetic resonance imaging in murine Chagas disease. The American journal of tropical medicine and hygiene. 1999;61(2):207–14. 10463668 10.4269/ajtmh.1999.61.207

[35] Ma H, Wang J, Thomas DP, Tong C, Leng L, Wang W, et al Impaired macrophage migration inhibitory factor-AMP-activated protein kinase activation and ischemic recovery in the senescent heart. Circulation. 2010;122(3):282–92. PubMed Central PMCID: PMC2907453. 10.1161/CIRCULATIONAHA.110.953208 20606117 PMC2907453

[36] Chan AY, Dolinsky VW, Soltys CL, Viollet B, Baksh S, Light PE, et al Resveratrol inhibits cardiac hypertrophy via AMP-activated protein kinase and Akt. The Journal of biological chemistry. 2008;283(35):24194–201. PubMed Central PMCID: PMCPMC3259789. 10.1074/jbc.M802869200 18562309 PMC3259789

[37] Sin TK, Tam BT, Yung BY, Yip SP, Chan LW, Wong CS, et al Resveratrol protects against doxorubicin-induced cardiotoxicity in aged hearts through the SIRT1-USP7 axis. The Journal of physiology. 2015;593(8):1887–99. PubMed Central PMCID: PMC4405749. 10.1113/jphysiol.2014.270101 25665036 PMC4405749

[38] Martin-Montalvo A, Mercken EM, Mitchell SJ, Palacios HH, Mote PL, Scheibye-Knudsen M, et al Metformin improves healthspan and lifespan in mice. Nature communications. 2013;4:2192 PubMed Central PMCID: PMC3736576. 10.1038/ncomms3192 23900241 PMC3736576

[39] Baez A, Lo Presti MS, Rivarola HW, Mentesana GG, Pons P, Fretes R, et al Mitochondrial involvement in chronic chagasic cardiomyopathy. Transactions of the Royal Society of Tropical Medicine and Hygiene. 2011;105(5):239–46. 10.1016/j.trstmh.2011.01.007 21470646

[40] Guzman Mentesana G, Baez A, Cordoba R, Dominguez R, Lo Presti S, Rivarola W, et al [Role of mitochondria and reactive oxygen species in the progression of heart failure]. Rev Fac Cien Med Univ Nac Cordoba. 2010;67(4):150–8. 21843439

[41] Zhang L, He H, Balschi JA. Metformin and phenformin activate AMP-activated protein kinase in the heart by increasing cytosolic AMP concentration. American journal of physiology Heart and circulatory physiology. 2007;293(1):H457–66. 10.1152/ajpheart.00002.2007 17369473

[42] Gundewar S, Calvert JW, Jha S, Toedt-Pingel I, Ji SY, Nunez D, et al Activation of AMP-activated protein kinase by metformin improves left ventricular function and survival in heart failure. Circulation research. 2009;104(3):403–11. PubMed Central PMCID: PMC2709761. 10.1161/CIRCRESAHA.108.190918 19096023 PMC2709761

[43] Francischetti IM, Gordon E, Bizzarro B, Gera N, Andrade BB, Oliveira F, et al Tempol, an intracellular antioxidant, inhibits tissue factor expression, attenuates dendritic cell function, and is partially protective in a murine model of cerebral malaria. PloS one. 2014;9(2):e87140 PubMed Central PMCID: PMC3938406. 10.1371/journal.pone.0087140 24586264 PMC3938406

[44] Valera Vera EA, Saye M, Reigada C, Damasceno FS, Silber AM, Miranda MR, et al Resveratrol inhibits Trypanosoma cruzi arginine kinase and exerts a trypanocidal activity. Int J Biol Macromol. 2016;87:498–503. 10.1016/j.ijbiomac.2016.03.014 26976067

[45] Sasaki H, Asanuma H, Fujita M, Takahama H, Wakeno M, Ito S, et al Metformin prevents progression of heart failure in dogs: role of AMP-activated protein kinase. Circulation. 2009;119(19):2568–77. 10.1161/CIRCULATIONAHA.108.798561 19414638

[46] Britton RG, Kovoor C, Brown K. Direct molecular targets of resveratrol: identifying key interactions to unlock complex mechanisms. Annals of the New York Academy of Sciences. 2015;1348(1):124–33. 10.1111/nyas.12796 26099829

[47] Hao E, Lang F, Chen Y, Zhang H, Cong X, Shen X, et al Resveratrol alleviates endotoxin-induced myocardial toxicity via the Nrf2 transcription factor. PloS one. 2013;8(7):e69452 PubMed Central PMCID: PMC3718737. 10.1371/journal.pone.0069452 23894482 PMC3718737

[48] Park SJ, Ahmad F, Philp A, Baar K, Williams T, Luo H, et al Resveratrol ameliorates aging-related metabolic phenotypes by inhibiting cAMP phosphodiesterases. Cell. 2012;148(3):421–33. PubMed Central PMCID: PMC3431801. 10.1016/j.cell.2012.01.017 22304913 PMC3431801

[49] Price NL, Gomes AP, Ling AJ, Duarte FV, Martin-Montalvo A, North BJ, et al SIRT1 is required for AMPK activation and the beneficial effects of resveratrol on mitochondrial function. Cell metabolism. 2012;15(5):675–90. PubMed Central PMCID: PMC3545644. 10.1016/j.cmet.2012.04.003 22560220 PMC3545644

[50] Eickhoff CS, Lawrence CT, Sagartz JE, Bryant LA, Labovitz AJ, Gala SS, et al ECG detection of murine chagasic cardiomyopathy. The Journal of parasitology. 2010;96(4):758–64. PubMed Central PMCID: PMC3150498. 10.1645/GE-2396.1 20738200 PMC3150498

[51] Johnson JJ, Nihal M, Siddiqui IA, Scarlett CO, Bailey HH, Mukhtar H, et al Enhancing the bioavailability of resveratrol by combining it with piperine. Molecular nutrition & food research. 2011;55(8):1169–76. PubMed Central PMCID: PMCPMC3295233. 21714124 10.1002/mnfr.201100117 PMC3295233

