# Supplementary figures and images for: Resveratrol Reverses Functional Chagas Heart Disease in Mice
Source: PLoS Pathog. 2016 Oct 27;12(10):e1005947. doi: 10.1371/journal.ppat.1005947 (PMC5082855; doi:10.1371/journal.ppat.1005947)

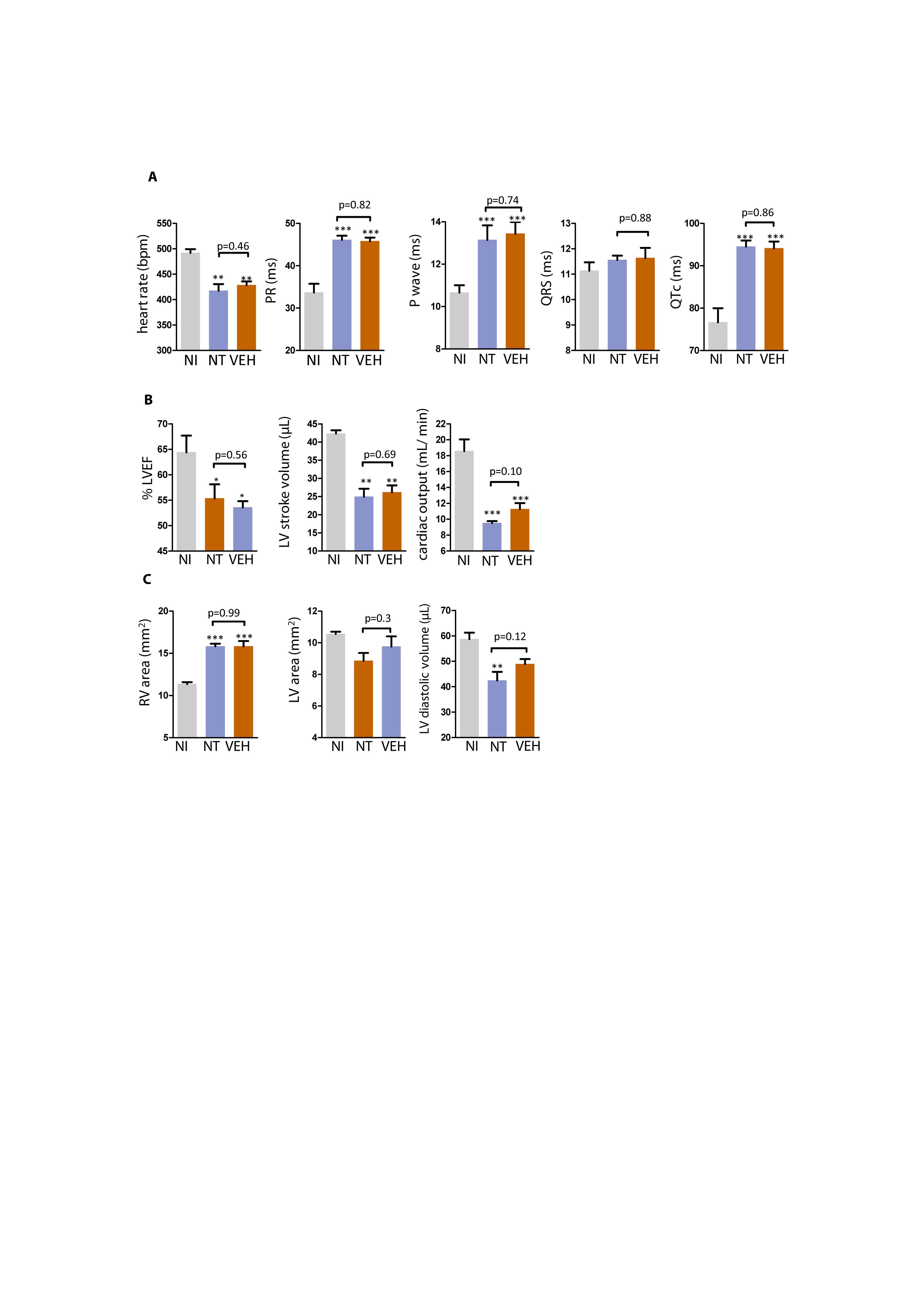

Supplement: S1 Fig — (A), ECG intervals at 90 dpi for non-infected (NI), infected non-treated (NT), or (VEH)-treated mice. (B) Left ventricle ejection fraction (LVEF), left ventricle stroke volume, cardiac output; (C) right ventricle area (RV area), left ventricle area (LV area). NI (n = 4), NT (n = 5), VEH (n = 6), representative of two independent experiments. Error bars indicate mean±SEM. *, different from NI. P range: *, P≤0.05, **, P<0.01, ***, P<0.005. (TIF) [file ppat.1005947.s001.tif]

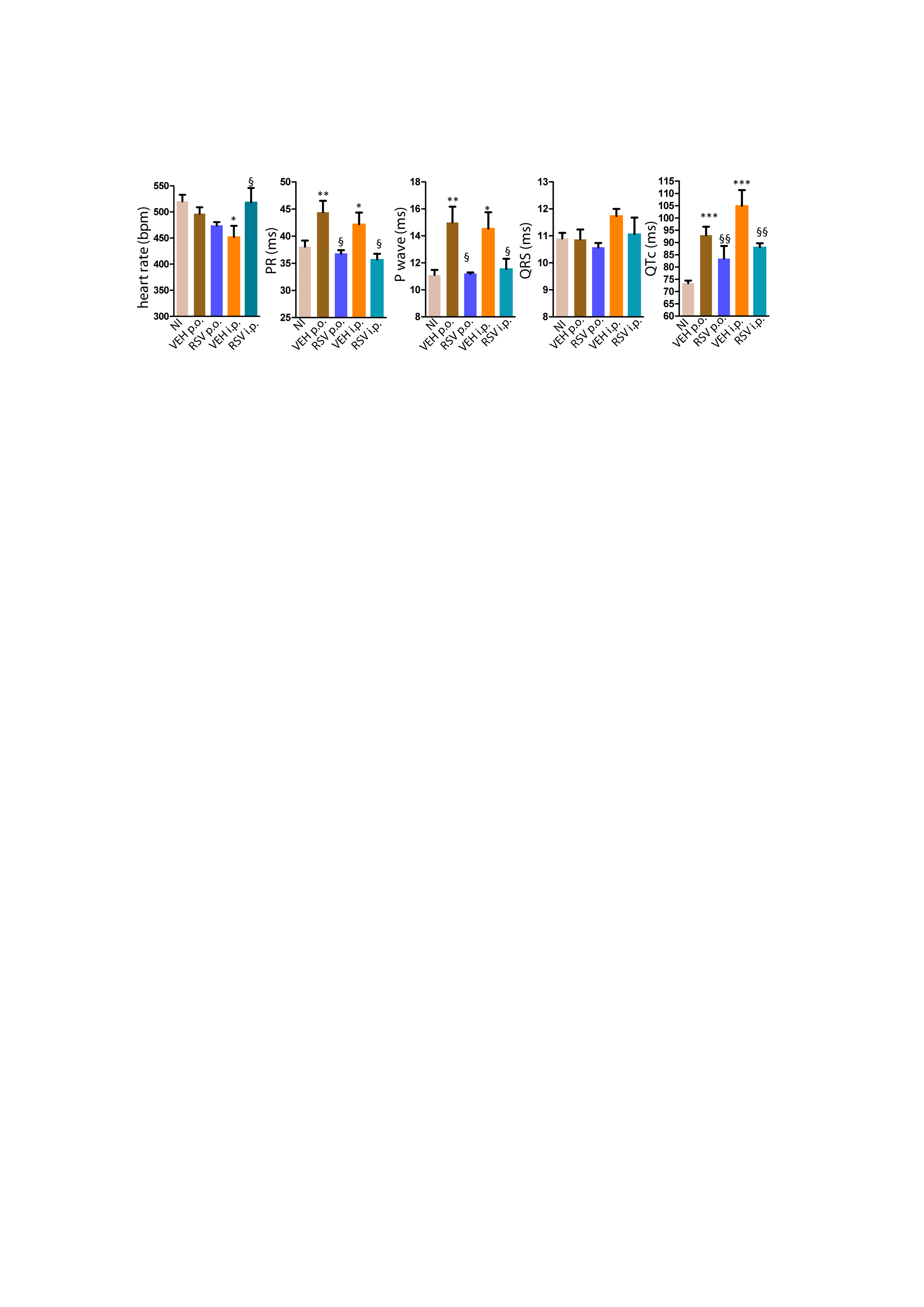

Supplement: S2 Fig — routes. ECG intervals at 90 dpi for noninfected (NI), infected peroral vehicle-treated (VEH p.o.), infected peroral resveratrol-treated (RSV p.o.), infected intraperitoneal vehicle-treated (VEH i.p.), infected intraperitoneal resveratrol-treated mice (RSV i.p.) NI (n = 9), VEH p.o. (n = 8), RSV p.o. (n = 4), VEH i.p. (n = 4), RSV i.p. (n = 5). Error bars indicate mean±SEM. *, different from NI; §, different from respective VEH. P range: * §, P≤0.05, ** §§, P<0.01, ***, P<0.001 (TIF) [file ppat.1005947.s002.tif]

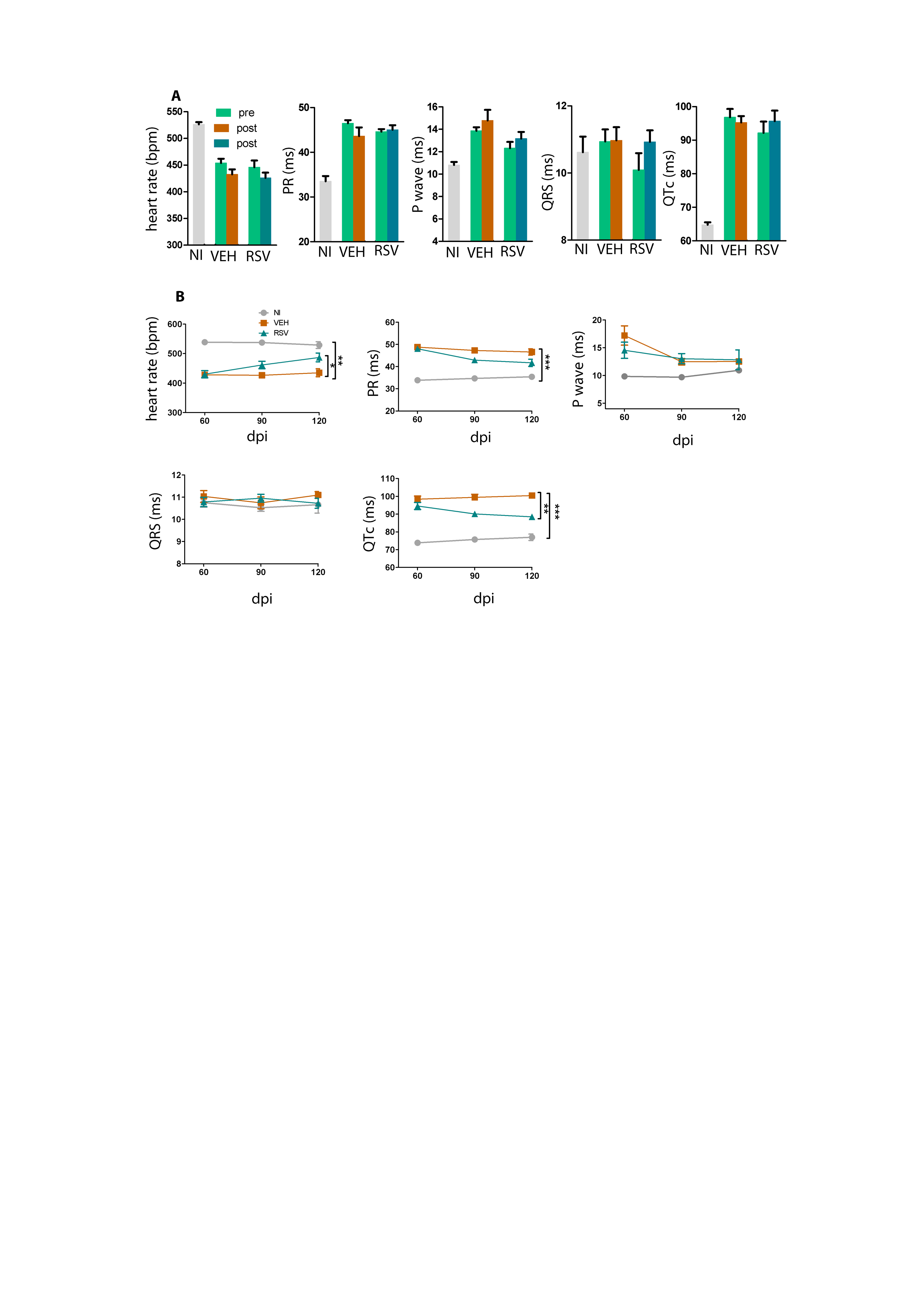

Supplement: S3 Fig — (A), ECG intervals before and 20 h after treatment of infected mice (60 dpi) with vehicle (VEH) or resveratrol (RSV). Mice per group: NI (n = 5), VEH (n = 10), and RSV (n = 10). (B), ECG intervals before (60 dpi) and throughout treatment (90 dpi, 120 dpi) of infected mice with vehicle or resveratrol. Mice per group: NI (n = 4–10), VEH (n = 4–10), RSV (n = 8–10). A physiological zero was adopted on the Y axis (lowest value found by us in mice). The Pvalue was calculated using ANOVA to compare groups at 120 dpi. Error bars indicate mean±SEM. *, different from NI; §, different from VEH. P range: §, P≤0.05, **, P<0.01, ***, P<0.005 (TIF) [file ppat.1005947.s003.tif]

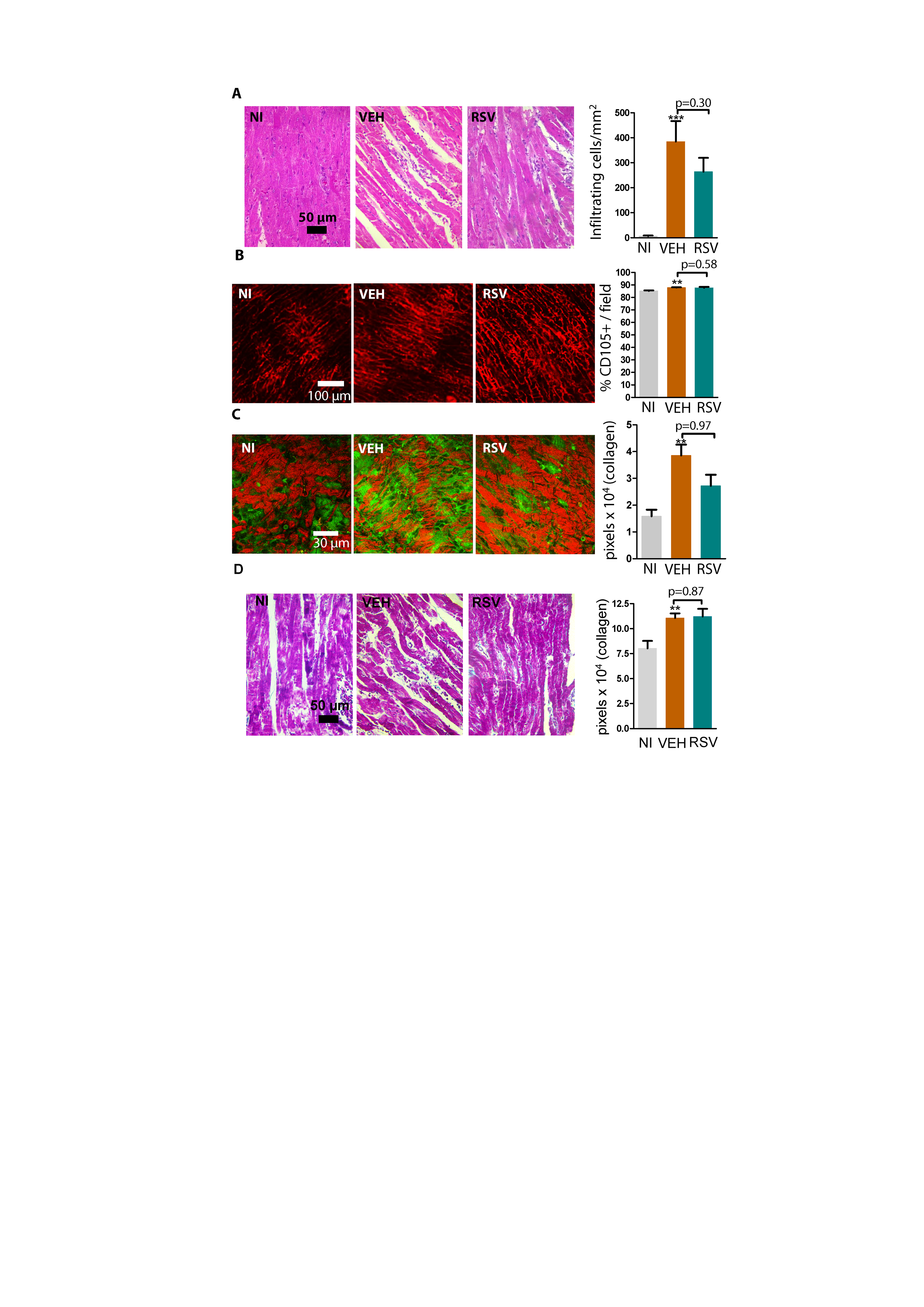

Supplement: S6 Fig — Heart histology was assessed 30 days after treatment of infected mice with either vehicle or resveratrol (at 90 dpi). (A) H&E heart slides and graph showing inflammatory infiltrates in the hearts of infected vehicle (VEH)- or resveratrol (RSV)-treated mice. No differences were found among ventricles. Hearts per group: NI (n = 8), VEH (n = 13), and RSV (n = 10), pooled from 2 independent experiments. (B) Confocal microscopy of fresh heart explants from mice injected in vivo with labeled anti-CD105 to reveal blood vessels, n = 3 hearts / group. (C) 2nd harmonic imaging microscopy of fixed heart explants to reveal collagen (green) and structural myocardium proteins (red). Interstitial collagen was estimated from these images. Hearts per group: NI (n = 3), VEH (n = 5) and RSV (n = 5). (D) 3-color Masson was used to reveal interstitial collagen in left ventricle. No significant differences were found among VEH and RSV ventricles. n = 9 hearts / group. Error bars indicate mean±SEM. *, different from NI. P range: **, P<0.01, ***, P<0.005. (TIF) [file ppat.1005947.s006.tif]

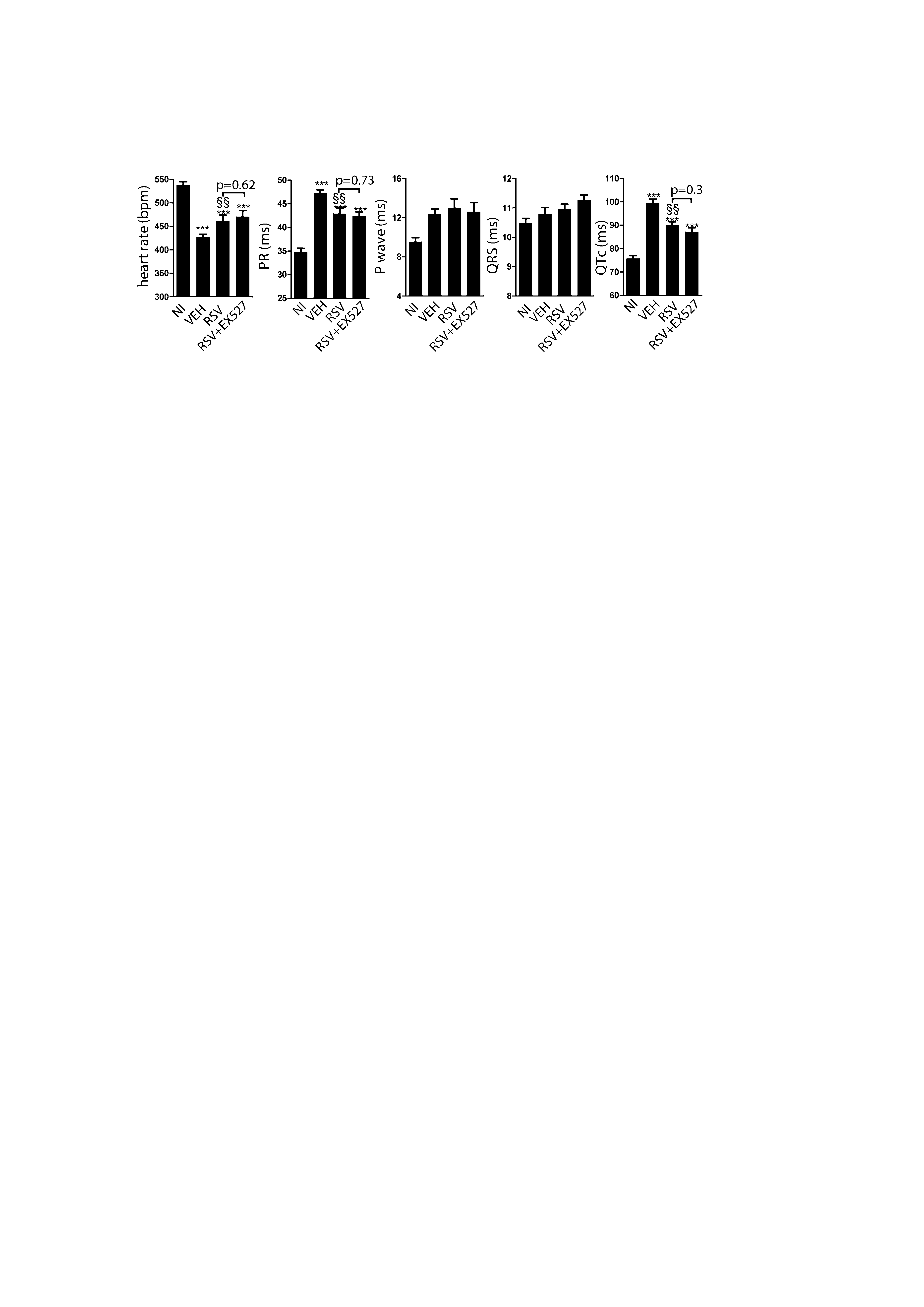

Supplement: S7 Fig — Infected mice were treated with either resveratrol (RSV) or vehicle (VEH) from 60–90 dpi and their heart electrical function was assessed by ECG at 90 dpi. NI (n = 5), VEH (n = 5), RSV (n = 7). Error bars indicate mean±SEM. NI n = 11, VEH n = 10, RSV n = 13, EX527+RSV n = 9. *, different from NI; §, different from VEH. P range: *, § P≤0.05, ** §§, P<0.01, ***, P<0.005. (TIF) [file ppat.1005947.s007.tif]

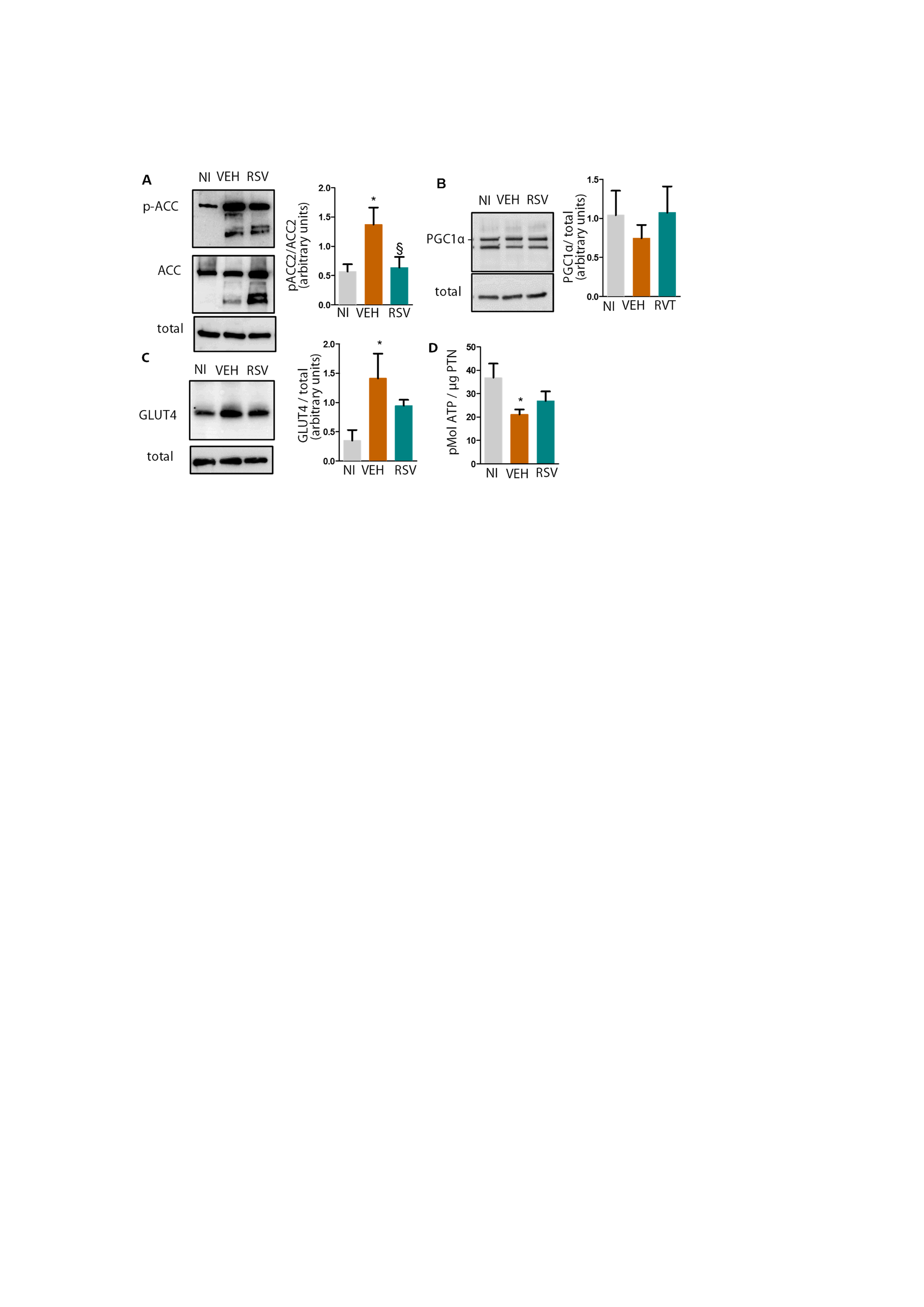

Supplement: S8 Fig — (A) Ventricle extracts were probed 30 days after treatment of infected mice (with either vehicle or resveratrol) with antibodies against ACC (ACC2 is the top band), p-ACC, and constitutive control (total). Results are shown for ACC2. Sum of three independent experiments (n = 9 hearts per group). (B) Similar to (A), individual ventricle extracts were probed for PGC1α (top band). Hearts per group: NI n = 5, VEH n = 6, RSV n = 6 (sum of two independent experiments) or (C) GLUT4 (n = 3 hearts per group). (D) ATP amounts in ventricle extracts. Pooled from two independent experiments (NI n = 5, VEH n = 6, RSV n = 5 hearts per group). Error bars indicate mean±SEM. *, different from NI; §, different from VEH. P range: *, § P≤0.05. (TIF) [file ppat.1005947.s008.tif]

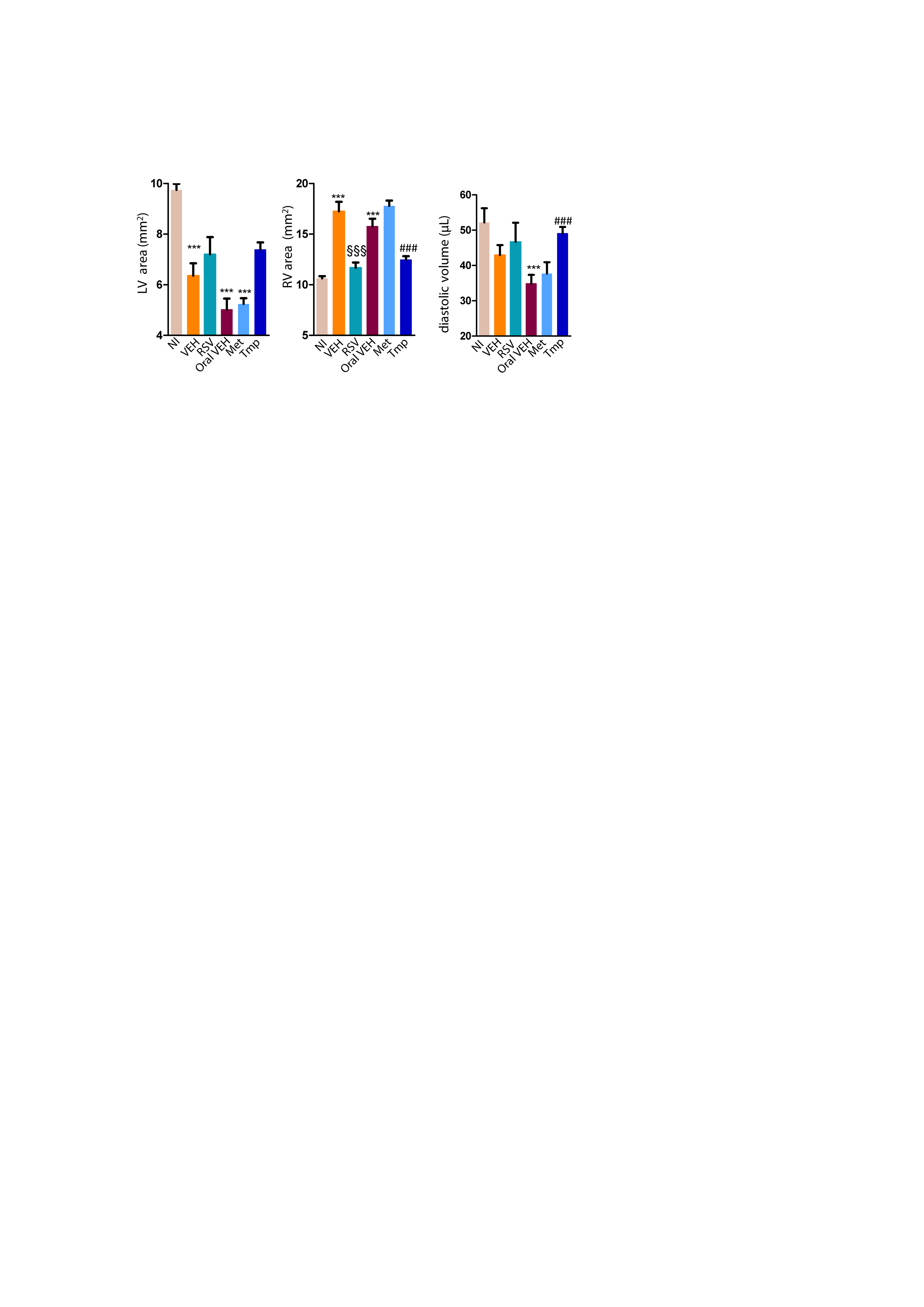

Supplement: S9 Fig — Infected mice were treated with peroral metformin (Met), tempol (Tmp), or oral vehicle (VEH) from 60–90 dpi. Infected mice treated i.p. with resveratrol (RSV) or vehicle (VEH) were kept as controls, as well as non-infected mice. Left ventricle area, right ventricle area and left ventricle diastolic volume. Mice per group: NI (n = 11), VEH (n = 7), Met (n = 9), Tmp (n = 9). Met are representative of 2 similar experiments. *, different from NI; §, different from VEH, #, different from oral VEH. P range: *, P≤0.05, **, P<0.01, *** §§§ ###, P<0.005 (TIF) [file ppat.1005947.s009.tif]
