# Supplementary material for: Resveratrol Reverses Functional Chagas Heart Disease in Mice
Source: PLoS Pathog. 2016 Oct 27;12(10):e1005947. doi: 10.1371/journal.ppat.1005947 (PMC5082855; doi:10.1371/journal.ppat.1005947)
Supplement: S1 Text — (DOCX) [file ppat.1005947.s010.docx]

**Online Methods:**

**Electrocardiography (ECG).** The QT end was determined taking the end of the T wave, determined as the point at which the component of the T wave merges with the isoelectric baseline. We corrected the QT by the heart rate to obtain the QTc. The corrected QT (QTc) was calculated from the QT (QT0) using a RR interval (RR0) normalized to a multiple of 100ms (RR100) and applying the formula: QT0=ln (QTc)+y ln RR100. The exponent y is the slope of the linear relationship between ln QT and RR100, to which the RR interval should be raised in order to correct QT for heart rate. We adopted a physiologic rather than a numeric zero as the basal value for the Y axis of electro and echocardiographs. The physiologic zero was determined by the smaller value observed in living mice. The magnitude of the variations among groups is compared within the vital range.

**Lipid peroxidation.** To assess lipid peroxidation, we dosed the byproduct malondialdehyde by reacting the samples with thiobarbituric acid (Sigma). Thiobarbituric acid reactive substances (TBARS) were determined spectrophotometrically in serum samples and cardiac tissue. Briefly, the serum and tissue homogenates prepared in 0.9% NaCl were mixed with 8.1% sodium dodecylsulfate (SDS), pH 3.5 acetic acid solution and 0.8% thiobarbituric acid. The mixture was incubated for 50 min at 95°C followed by the addition of n-butanol and centrifugation at 4000 rpm for 10 min. The absorbance was determined at 532 nm.

**Quantitative Polymerase Chain Reaction (qPCR) for parasite detection.** Heart tissue samples weighing 10 mg were submitted to DNA extraction according to the manufacturer’s instructions (DNeasy Blood & Tissue Kit – Qiagen). Genomic DNA samples were visualized by agarose gel electrophoresis and submitted to real time PCR using the specific oligonucleotides (mmGAPDH-F 5’

AACTTTGGCATTGTGGAAGG 3’, mmGAPDH-R 5’ ACACATTGGGGGTAGGAACA 3’, TCZ-F 5’ GCTCTTGCCCACAAGGGTGC 3’, TCZ-R 5’ CCAAGCAGCGGATAGTTCAGG 3’). Power SYBR

Green 1X (Applied Biosystems) and 200nM of each oligonucleotide were used for amplification in an ABI PRISM 7500 Sequence Detection System thermocycler (Applied Biosystems), with the following cycle: denaturation in 95°C for 10 minutes, 40 cycles at 95°C for 15 sec, and 60°C for 1 min. Then, a denaturation curve was built to verify the presence of non-specific products. Oligonucleotide efficiency was analyzed by serial dilution and ranged between 97% and 98%. The relative quantification of the target genes was determined using the comparison Ct method.

**Western blot.** Protein extracts were obtained by homogenizing 100 mg of heart ventricle tissue in lysis buffer (Sacarose 0.32M, EDTA 1mM, EGTA 1mM, Tris pH 7.4 10mM and protease inhibitor cocktail – Sigma). After 30 min in ice, the samples were submitted to centrifugation for 20 min at 20,000g and 4°C. Samples were quantified by Bradford’s method (BIO-RAD), divided in aliquots, and stored at -80°C. Protein extracts were submitted to denaturing polyacrylamide gel electrophoresis (Any kD™ or 4-15% Mini-PROTEAN® TGX™ - Bio-Rad) and transferred onto nitrocellulose membranes (Millipore). Membranes were blocked (TBS 1X, 0.05% Tween 20, and 5% BSA) for 1h at room temperature and then incubated overnight at 4°C with protein-specific primary antibodies. Membranes were then washed 3x in TBS 1X 0.05% tween (washing buffer) and incubated with secondary antibodies diluted at 1:10,000 for 1 h at room temperature. Finally, the membranes were washed and proteins were detected by chemiluminescence using DuraECL (Amersham). Images were acquired with ImageQuant LAS 4000 (GE). Primary antibodies: pAMPK Thr172 (Cell Signalling, 4188S), AMPK (cs 2532S), pACC Ser79 (cs 11818), ACC (cs 3676), p38 (cs 9212S), GLUT4 (cs 22135), PGC1a (Abcam, ab72239), SIRT1 (ab156065), SOD2 (ab13533). Secondary antibodies: anti-mouse IgG (Thermo Scientific 31430); anti-rabbit IgG (KPL 04-15-06). We tested β-actin, tubulin, and GADPH as possible constitutive loading controls, but they did change their levels of expression during infection, so we chose p38 as a more stable constitutive control (total protein levels).

**Ex-vivo heart confocal microscopy for ROS and vessels**. Total- and mitochondrial-ROS production were evaluated by laser confocal microscopy of heart explants. Mice were intravenously injected with 2µg APC anti-CD31 (eBioscience 17-0311) and PE anti-CD105 (eBioscience 12-1051), and either 15 mg/mice H_2_DCFDA (Molecular Probes D399), for total ROS production, or 500nmol/kg MitoSOX (Molecular Probes M36008), for mitochondrial ROS. Probes and antibodies were allowed to circulate for 15 minutes and mice were sacrificed by isofluorane overdose. Hearts were then dissected and the left ventricles were excised and immediately analyzed by confocal microscopy using a Zeiss LSM 510 Microscope equipped with a 488nm, a 561nm, and a 633nm laser and a 20X/0.50 NA EC Plan-NeoFluor objective. Images were acquired using a 488/561/633 HFT excitation filter (for all channels), together with a 500-520nm BP filter (for green channel), a 575-615nm BP filter (for the red channel) and a 650-710nm BP filter (for the far-red channel), with the help of ZEN 1000 software. At least six pictures were analyzed per mouse heart (apex). Acquired images were analyzed with ImageJ software to determine mean fluorescence intensity (ROS probes) or stained area (vessels).

**Second-harmonic generation for collagen detection**. To analyze heart interstitial fibrosis, we evaluated collagen deposition by second harmonic generation (SHG). Mice were sacrificed and the left ventricle was excited and fixed in buffered formaldehyde. Tissue samples were analyzed on a

Zeiss LSM 510 microscope equipped with a MaiTai laser and a 63X/1.40 NA Plan-Apochromat Oilimmersion objective. Second harmonic was generated at 820nm excitation and detected with a 390465nm BP filter. For reference, tissue autofluorescence was also acquired at 820nm excitation, with a 500-550nm filter. Stack images were acquired at a 5µm interval from tissue capsule (denoted as zero) up to 35-40µm deep. At least 4 pictures were analysed per mouse heart. Acquired images were analyzed with ImageJ softwareto determine the area of collagen.

**Histopathological studies.** Hearts were collected, fixed in formaldehyde, embedded in paraffin, and cut in transverse 5 μM sections, mounted on slides, and stained on a standard H&E or trichrome Masson technique. Heart transverse sections were randomly photographed (right and left ventricles) under an Olympus C21 microscope equipped with a Leica DFC495 camera. To assess inflammatory infiltrates, pictures were printed and infiltrating inflammatory cells were counted on 20 pictures taken from each individual heart. To evaluate the diameter of heart cardiomyocytes, we analyzed longitudinal ventricular fibers in trichrome Masson-stained sections using ImageJ. At least 50 fibers were analyzed per mouse slide.

**SIRT1 deacetylase activity.** Heart lysates were prepared and SIRT1 activity was determined in 10μL samples using an Abcam kit, ab156065, according to the manufacturer’s instructions. Controls were prepared for each extract omitting NAD addition and discounted from the total extract fluorescence. Positive controls were prepared with 125 ng of purified SIRT1, producing approximately 10x the activity found in extracts. All fluorescence readings (Spectramax I3, Molecular Devices) were normalized to the respective protein concentrations.

**ATP dosage.** Heart samples were homogenized 1:10 (w/v) in buffer containing 50 mM Tris-HCl pH 7.2 100 mM KCl 1 mM EDTA 2% de TCA. Protein concentration in aliquots taken from those extracts was measured using Bradford’s method (Bradford reagent, Sigma). A pre-heated (95°C) extraction buffer (200 mM Tris-HCl pH 7.75 4 mM EDTA) was added to the homogenate 1:9 (v/v). Samples were boiled for 1 min and then transferred to an ice bath for 5 min. The samples were then centrifuged (1000 g for 5 min) and the supernatant was used for quantification of ATP in heart samples using ATP Bioluminescent Assay Kit– FL-AA (Sigma), according to the manufacturer’s instructions. We built a linear standard curve using 25 pM-25 nM ATP and the ATP amounts were normalized to the protein concentration in each heart sample to obtain pM ATP/µg of heart protein.
